# A hypermethylation strategy utilized by enhancer-bound CARM1 to promote estrogen receptor α-dependent transcriptional activation and breast carcinogenesis

**DOI:** 10.7150/thno.39241

**Published:** 2020-02-10

**Authors:** Bing-ling Peng, Wen-juan Li, Jian-cheng Ding, Yao-hui He, Ting Ran, Bing-lan Xie, Zi-rui Wang, Hai-feng Shen, Rong-quan Xiao, Wei-wei Gao, Tian-yi Ye, Xiang Gao, Wen Liu

**Affiliations:** 1 Fujian Provincial Key Laboratory of Innovative Drug Target Research, School of Pharmaceutical Sciences, Xiamen University, Xiang'an South Road, Xiamen, Fujian 361102, China; 2 State Key Laboratory of Cellular Stress Biology, School of Pharmaceutical Sciences, Xiamen University, Xiang'an South Road, Xiamen, Fujian 361102, China

**Keywords:** Protein arginine methyltransferase, protein arginine methylation, tudor domain-containing protein, estrogen receptor, breast cancer

## Abstract

While protein arginine methyltransferases (PRMTs) and PRMT-catalyzed protein methylation have been well-known to be involved in a myriad of biological processes, their functions and the underlying molecular mechanisms in cancers, particularly in estrogen receptor alpha (ERα)-positive breast cancers, remain incompletely understood. Here we focused on investigating PRMT4 (also called coactivator associated arginine methyltransferase 1, CARM1) in ERα-positive breast cancers due to its high expression and the associated poor prognosis.

**Methods**: ChIP-seq and RNA-seq were employed to identify the chromatin-binding landscape and transcriptional targets of CARM1, respectively, in the presence of estrogen in ERα-positive MCF7 breast cancer cells. High-resolution mass spectrometry analysis of enriched peptides from anti-monomethyl- and anti-asymmetric dimethyl-arginine antibodies in SILAC labeled wild-type and CARM1 knockout cells were performed to globally map CARM1 methylation substrates. Cell viability was measured by MTS and colony formation assay, and cell cycle was measured by FACS analysis. Cell migration and invasion capacities were examined by wound-healing and trans-well assay, respectively. Xenograft assay was used to analyze tumor growth *in vivo*.

**Results**: CARM1 was found to be predominantly and specifically recruited to ERα-bound active enhancers and essential for the transcriptional activation of cognate estrogen-induced genes in response to estrogen treatment. Global mapping of CARM1 substrates revealed that CARM1 methylated a large cohort of proteins with diverse biological functions, including regulation of intracellular estrogen receptor-mediated signaling, chromatin organization and chromatin remodeling. A large number of CARM1 substrates were found to be exclusively hypermethylated by CARM1 on a cluster of arginine residues. Exemplified by MED12, hypermethylation of these proteins by CARM1 served as a molecular beacon for recruiting coactivator protein, tudor-domain-containing protein 3 (TDRD3), to CARM1-bound active enhancers to activate estrogen/ERα-target genes. In consistent with its critical role in estrogen/ERα-induced gene transcriptional activation, CARM1 was found to promote cell proliferation of ERα-positive breast cancer cells *in vitro* and tumor growth in mice.

**Conclusions**: our study uncovered a “hypermethylation” strategy utilized by enhancer-bound CARM1 in gene transcriptional regulation, and suggested that CARM1 can server as a therapeutic target for breast cancer treatment.

## Introduction

Based on the expression of estrogen receptor (ER), progesterone receptor (PR) and human epidermal growth factor receptor 2 (HER2), breast cancer could be classified into at least five subtypes: luminal A, luminal B, HER2, triple-negative and normal-like 1, 2. Luminal A and B subtypes together constitute the so-called ER-positive breast cancers, which account for around 70% of all diagnosed patients. Prolonged exposure to high levels of the steroid hormone estrogens (17-β-estradiol, estradiol, E_2_) has been shown to be a major cause of ER-positive breast cancers, which constitutively activates the transcription of genes predominantly implicated in metabolism and cell cycle regulation. Steroid hormone estrogen's effects on breast tumorigenesis are mediated through its receptor, ER, which belongs to a superfamily of nuclear receptors that act as ligand-dependent transcriptional factors. ER is activated upon binding of steroid estrogen hormones, translocating from cytosol to nucleus and recruiting a plethora of co-activators to its target gene promoter and/or enhancer regions to regulate gene transcription 3, 4. Among these co-regulators, a group of proteins called epigenetic enzymes are of particular interest, which possess enzymatic activities to regulate histone modifications and chromatin remodeling, and thus estrogen/ER-mediated transcriptional activation 5, 6.

PRMT (protein arginine methyltransferase) protein family has been shown to include at least nine members in mammalian genomes, designated as PRMT 1 to 9. PRMTs transfer a methyl group from S-adenosyl-L-methionine (SAM) to the side chain of arginine residues in proteins, resulting in the formation of three types of final products: ω-N^G^-mono-methylated arginine (MMA), ω-N^G^, N^G^-asymmetric dimethyl-arginine (ADMA) and ω- N^G^, N'^G^-symmetric dimethyl-arginine (SDMA). Depending on the form of methylated arginine PRMTs can catalyze, PRMTs are classified into three major types, in which type I PRMTs, including PRMT1, PRMT2, PRMT3, PRMT4/CARM1, PRMT6 and PRMT8, catalyze both MMA and ADMA, type II PRMTs, including PRMT5 and PRMT9, catalyze both MMA and SDMA, and type III PRMT, PRMT7, catalyzes only MMA. While protein arginine methylation does not alter amino-acid charge, it does increase its bulkiness and hydrophobicity, leading to the change of higher order protein structure and thus protein-nucleic acid and protein-protein interaction 7. A recent study revealed that around 7% of all arginine residues in the proteome are methylated, which is comparable to 9% of serine residues being phosphorylated and 7% of lysine residues being ubiquitinated 8. Arginine methylation has been identified in proteins with localization ranging from cell membrane, cytosol to nuclei 8-12, and it has been implicated in myriad biological pathways, including transcription, RNA processing and transport, translation, protein stability, signal transduction and DNA repair, among others 13, 14. Aberrant expression and/or activity of multiple PRMTs have been linked to human diseases, including cancers, cardiovascular diseases, viral pathogenesis and spinal muscular atrophy, among others 15. Particularly, the role of PRMTs in cancers has been well documented 16, 17.

PRMT4, also known as CARM1 (coactivator associated arginine methyltransferase 1), is a type I arginine methyltransferase, which adds both mono- and asymmetric di-methylation to arginine residues in histone H3 as well as an ever expanding number of non-histone proteins 8, 9, 18. CARM1 has indispensable roles in embryonic development and cellular differentiation 19-21. Emerging evidence also supports the pathological roles of CARM1 in human disease, particularly in cancer. Increased CARM1 expression and/or activity has been reported in a variety of cancer types, including breast, prostate, colorectal, lung and liver cancer, and its higher expression often correlates with poor prognosis 22-30. CARM1 is thought to contribute to cancer progression mainly through its coactivator activity targeting a plethora of transcription factors, such as p53, E2F1 and NFκB, and/or its methyltransferase activity targeting oncogenic proteins, such as BAF155, NCOA3 and PKM2 25, 31-35. Among all cancer types, the role of CARM1 in breast cancer, particularly in ER-positive breast cancer, was most studied 18, 25, 27, 29, 30, 33, 36-43. However, the molecular mechanisms underlying CARM1 regulation of estrogen/ER-mediated gene transcriptional activation remain incompletely understood, which is partially due to lack of genome- and proteome-wide study to systematically reveal their genomic binding sites, transcriptional targets and cancer-relevant substrates.

Tudor family is the major protein domain family known to recognize methyl-arginine motif so far. Tudor domains are roughly sixty amino acids in length, which fold into four antiparallel β-strands 44. The first tudor-domain containing protein reported to bind with methylated arginine motif was human survival motor neuron (SMN) protein 45, 46. In humans, there are at least thirty-six proteins that harbor one or more tudor domains, among which several have been clearly shown to recognize methyl-arginine, including SMN, SPF30, TDRD1, TDRKH/TDRD2, TDRD3, TDRD6, TDRD9 and SND1/TDRD11 47. TDRD3 and SND1/TDRD11 are known to regulate gene transcription, whereas SMN and SPF30 are implicated in the regulation of splicing. TDRD1, TDRKH/TDRD2, TDRD6 and TDRD9 are uniquely participated in a gonad-specific small RNA silencing pathway 47. Notably, the tudor-domain of TDRD3 was shown to recognize H4R3me2a (asymmetrical di-methylated arginine 3 in Histone H4), H3R17me2a as well as a methylated arginine (R1810) in the C-terminal domain (CTD) of RNA polymerase II (RNA Pol II) mediated by CARM1, exhibiting transcriptional coactivator activity 41, 48. However, whether TDRD3 also reads CARM1-mediated methylation on other substrates, and whether this recognition by TDRD3 participates in estrogen-induced transcriptional activation remains unknown.

In the current study, employing genomic and transcriptomic approaches, we uncovered the chromatin-binding landscape and transcriptional targets of CARM1 in the presence of estrogen in ERα-positive breast cancer cells. CARM1 was found to be predominantly and specifically recruited to ERα-bound active enhancers and essential for the transcriptional activation of cognate estrogen-induced gene. To further explore the molecular mechanisms underlying CARM1 regulation of estrogen-induced transcriptional activation, we focused on investigating CARM1-catalyzed methylation in the proteome by quantitative mass spectrometry analysis in wild-type (WT) and CARM1 knockout (KO) MCF7 cells. Notably, a large number of arginine residues (> 700) in proteins with diverse functions were exclusively methylated by CARM1, and, intriguingly, a number of proteins were found to be exclusively hypermethylated by CARM1 on a cluster of arginine residues. Exemplified by MED12, a component in the mediator complex, hypermethylation of these proteins by CARM1 served as a molecular beacon for recruiting tudor-domain containing protein TDRD3 to activate estrogen/ERα target genes. In consistent with its critical role in estrogen/ERα-induced gene transcriptional activation, CARM1 was found to promote cell proliferation of ERα-positive MCF7 breast cancer cells *in vitro* and tumor growth in mice.

## Results

### CARM1 is required for estrogen-induced gene transcriptional activation

**Figure 1:**
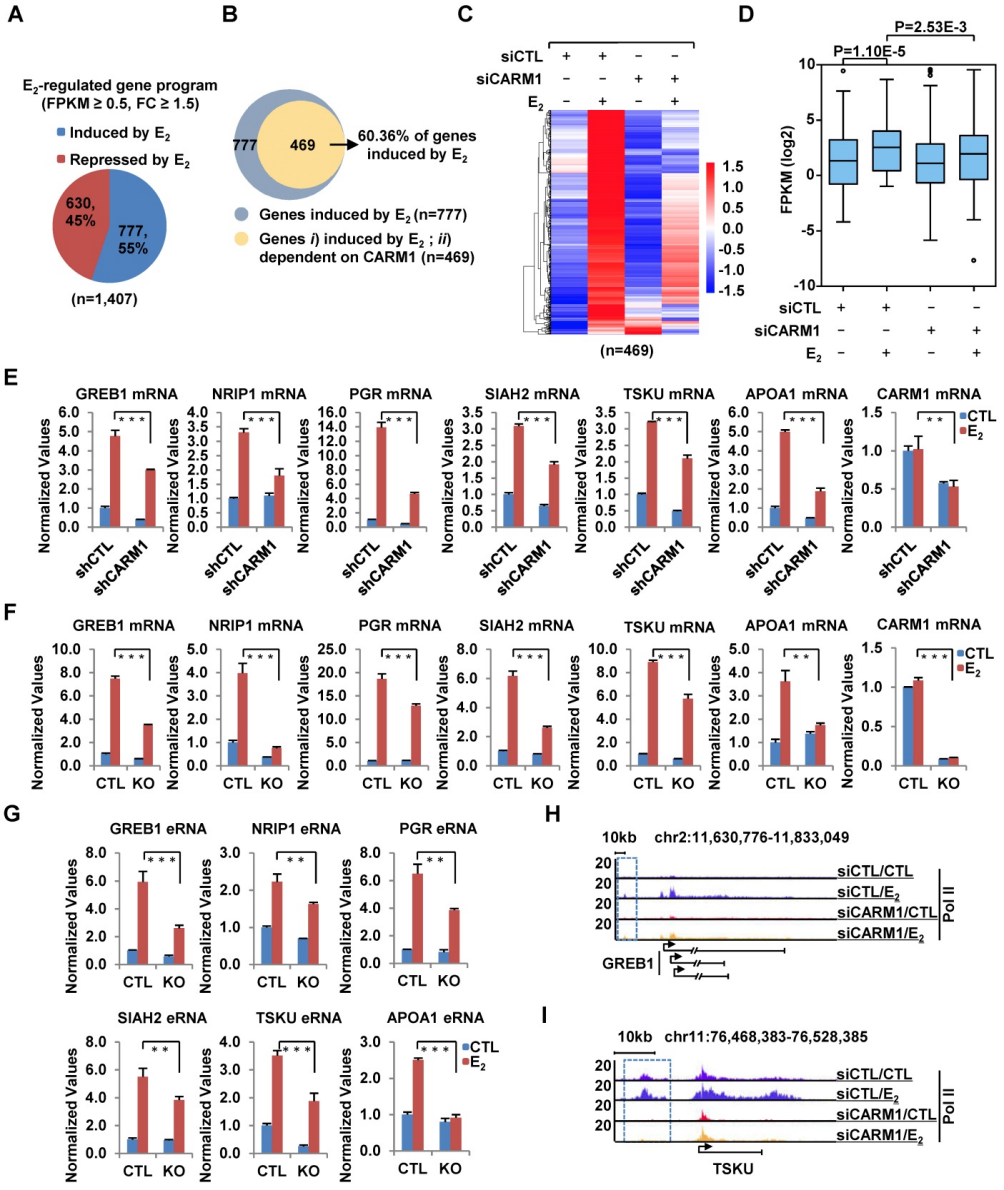
** CARM1 is required for estrogen-induced gene transcriptional activation.** (A) MCF7 cells were transfected with control siRNA (siCTL) or siRNA specific against CARM1 (siCARM1) in stripping medium for three days, and then treated with or without estrogen (E_2_, 10^-7^ M, 6 hr) followed by RNA-seq. Genes regulated by estrogen were shown (fold change (FC) (siCTL (E_2_)/siCTL (CTL)) ≥ 1.5). (B) Venn diagram showing genes induced by estrogen and dependent on CARM1 for expression (fold change (FC) (siCTL (E_2_)/siCARM1 (E_2_)) ≥ 1.5). (C, D) Heat map (C) and box plot (D) representation of the expression levels for genes induced by estrogen and dependent on CARM1 as described in (B). Heat map: z-score normalized FPKM; box plot: FPKM (log2). (E) MCF7 cells were infected with control shRNA (shCTL) or shRNA specific against CARM1 (shCARM1) in stripping medium for three days, and treated with or without estrogen (E_2_, 10^-7^ M, 6 hrs), followed by RNA extraction and RT-qPCR analysis to examine the expression of selected estrogen-induced genes as indicated (± s.e.m., **P<0.01, ***P<0.001). (F, G) Wild type (WT) and CARM1 knockout (KO) MCF7 cells were maintained in stripping medium for three days before treating with or without estrogen (E_2_, 10^-7^ M, 6 hrs), followed by RNA extraction and RT-qPCR analysis to examine the expression of selected estrogen-induced genes (F) and cognate enhancer RNAs (eRNAs) (G) as indicated (± s.e.m., **P<0.01, ***P<0.001). (H, I) MCF7 cells were transfected with siCTL or siCARM1 in stripping medium for three days, and treated with or without estrogen (E_2_, 10^-7^ M, 1 hr) followed by RNA Pol II ChIP-seq analysis. The distribution of Pol II was shown for specific genes, as indicated. Boxed regions indicated cognate active enhancers (also see Figure 2H and 2I).

**Figure 2:**
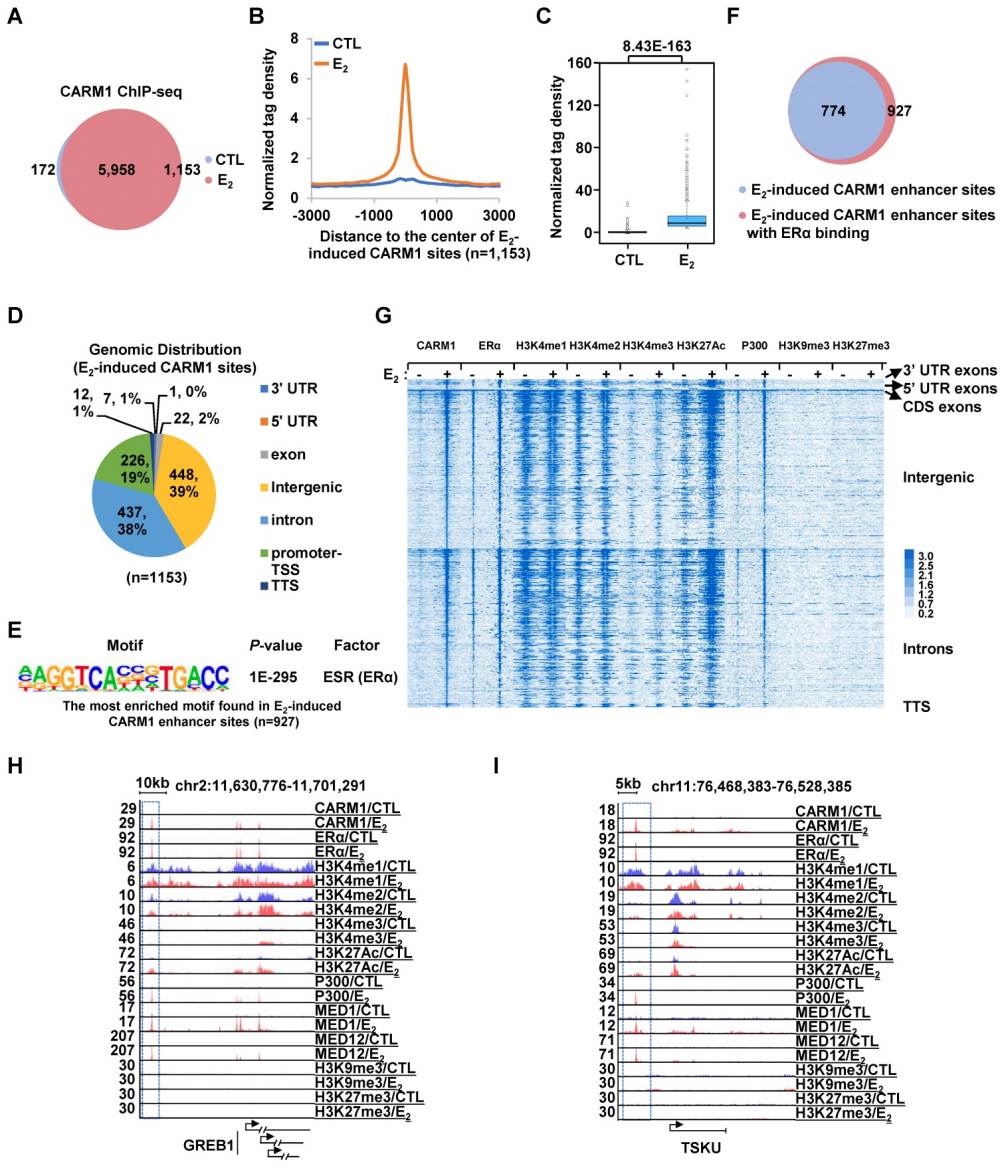
** CARM1 is recruited onto ERα-bound active enhancers in the presence of estrogen.** (A) MCF7 cells treated with or without estrogen (E_2_, 10^-7^ M, 1 hr) were subjected to ChIP-seq with anti-CARM1 specific antibody. CARM1 ChIP-seq binding sites in the presence or absence of estrogen was shown by venn diagram (Fold change (FC) (E_2_/CTL) larger than 4 was considered as E_2_ specific). (B) CARM1 ChIP-seq tag density distribution centered on estrogen-induced CARM1 sites (± 3,000 bp). (C) Box plot representation of the CARM1 ChIP-seq tag density on estrogen-induced CARM1 sites (± 3,000 bp). (D) Genomic distribution of estrogen-induced CARM1 sites. (E) Motif analysis of estrogen-induced CARM1 distal sites. (F) Pie chart showing estrogen-induced CARM1 distal sites with or without ERα. (G) Heat map representation of CARM1, ERα, H3K4me1, H3K4me2, H3K4me3, H3K27Ac, P300, MED1, H3K9me3 and H3K27me3 ChIP-seq tag density in the presence or absence of estrogen centered on estrogen-induced CARM1 distal sites (± 3,000 bp). (H, I) UCSC Genome browser views of CARM1, ERα, H3K4me1, H3K4me2, H3K4me3, H3K27Ac, p300, MED1, MED12, H3K9me3 and H3K27me3 ChIP-seq in the presence or absence of estrogen on selected active enhancer regions as indicated were shown. Boxed regions indicated cognate active enhancers. ChIP-seq views, except for CARM1, on *GREB1* have been shown in our previous study 54.

We compared the expression of CARM1 in a cohort of clinical breast tumor samples (n=1,102) to that of normal breast tissues (n=113) and found that its expression was significantly higher in tumors than normal tissues (**Figure S1A**). More importantly, Kaplan-Meier plotter analysis revealed that high expression of CARM1 correlates with poor prognosis (**Figure S1B** and **S1C**), which was consistent with previous report 29. These observations prompted us to investigate the potential role that CARM1 plays in breast carcinogenesis. We focused on studying the function of CARM1 in ERα-positive breast cancer in the current study, as which accounts for around 70% of all breast cancer patients. We first asked whether CARM1 is required for estrogen/ERα-induced gene transcriptional activation by transcriptomics analysis in MCF7, an ERα-positive breast cancer cell line. MCF7 cells were transfected with or without control siRNA (siCTL) or siRNAs specifically targeting CARM1 (siCARM1, also referred to siCARM1 (1)), treated with or without estrogen, and then subjected to RNA-seq analysis. Of a large cohort of 777 genes that were induced by estrogen (FC>1.5) (**Figure 1A**), expression of 469 of these genes was attenuated following knockdown of CARM1, representing nearly 61% of all estrogen-induced genes (**Figure 1B**). These 469 genes were referred to as estrogen-induced and CARM1-dependent genes. The expression of these 469 genes was shown by heat map (**Figure 1C**) and box plot (**Figure 1D**). CARM1's effects on representative estrogen-induced genes from RNA-seq, such as *TSKU* and *APOA1*, were shown (**Figure S1D** and** S1E**). Furthermore, the effects of CARM1 on estrogen response were highly reproducible between two biological repeats (**Figure S1F**) and between two independent siRNAs targeting CARM1 (siCARM1 (1) and siCARM1 (2)) (**Figure S1G**). The knockdown efficiency of the two siRNAs targeting CARM1 was examined by immunoblotting analysis (**Figure S1H**,** upper panel**). The expression of ERα was unaltered in the absence of estrogen when CARM1 was knocked down, as examined by both RT-qPCR and immunoblotting analysis. Interestingly, CARM1 knockdown seemed to attenuate estrogen-induced degradation of ERα (**Figure S1H**,** middle panel** and **S1I**). CARM1's positive effects on representative estrogen-induced gene transcriptional activation, such as *GREB1*, *NRIP1*, *PGR*, *SIAH2*, *TSKU* and *APOA1*, were confirmed by RT-qPCR analysis in MCF7 cells by lenti-viral shRNA (short hairpin RNA)-mediated knockdown. Meanwhile, genes such as *PRSS23*, *PDCD2L*, *PSAT1* and *CAD* were unaffected by CARM1 knockdown, which was consistent with RNA-seq analysis (**Figure 1E** and **Figure S1J**). The knockdown efficiency of shRNA targeting CARM1 was examined by immunoblotting analysis (**Figure S1K**). Furthermore, CARM1's effects on estrogen-induced gene transcriptional activation were confirmed in CARM1 knockout (KO) MCF7 cells (**Figure 1F**), which were generated by CRISPR (clustered, regularly interspaced, short palindromic repeats)/Cas9 system. One nucleotide insertion was found at the gRNA targeting region, which led to premature termination (**Figure S1L**). Knockout of CARM1 was confirmed by immunoblotting using two independent anti-CARM1 antibodies (**Figure S1M**). We also examined the expression of estrogen-induced enhancer RNAs (eRNAs) from enhancers corresponding to those estrogen-induced coding genes, and found that the production of eRNAs was significantly attenuated in CARM1 knockout cells (**Figure 1G,** see also Figure 2H and 2I). In consistent with its effects on estrogen-induced transcriptional activation, both coding genes and cognate enhancer RNAs, CARM1 knockdown led to a significant reduction of RNA Polymerase II (RNA Pol II) occupancy on those estrogen-induced and CARM1-dependent gene promoter and body regions as well as enhancer regions, such as *GREB1* and *TSKU* (**Figure 1H**,** 1I** and**Figure S1N-S1Q**). Significantly, the expression of those 469 genes that are estrogen-induced and CARM1-dependent was significantly higher in clinical breast tumor samples than normal breast tissues as mentioned above, suggesting that these genes might be clinically relevant (**Figure S1R** and **S1S**). Taken together, our data suggested that CARM1 is a critical regulator of estrogen-induced transcriptional activation, both enhancers and cognate coding genes.

### Estrogen induces CARM1 binding on ERα-bound active enhancers

Next, we sought to ask how CARM1 regulates estrogen-induced transcriptional activation. Several studies have suggested that CARM1 can bind to specific gene promoter and/or enhancer regions to regulate gene transcription 18, 25, 27, 29, 30, 33, 36-40, 42. To further uncover the genomic binding landscape of CARM1 in response to estrogen, we performed CARM1 ChIP-seq in MCF7 cells treated with or without estrogen. The specificity of the anti-CARM1 antibody used was evident from our immunoblotting analysis (**Figure S1H**, **S1K** and **S1M**). In the absence of regulatory signals, CARM1 binding sites were found to be localized on both promoter and enhancer regions. The top most enriched DNA motifs embedded in these CARM1-binding sites were those of Forkhead box proteins such as FOXA1, FOXM1, FOXA2 and FOXA3, AP-1/2 transcription factors such as FRA1, JUNB and FRA2, and GRHL2 (Grainyhead Like Transcription Factor 2). Upon estrogen treatment, there were additional 1,153 CARM1 binding sites that were strongly induced (fold induction (FC) > 4) (**Figure 2A-2C**). Nearly 80% of these estrogen-induced CARM1 binding sites were localized at distal regions (non-promoter regions) (n=927) (**Figure 2D**). Interestingly, there was often a distal CARM1-binding site in the vicinity of each of the rest 20% of promoter-occupied CARM1 sites, which might due to enhancer-promoter looping (*vide infra*). The binding of CARM1 was confirmed by ChIP-qPCR analysis (**Figure S2A**).

To characterize these estrogen-induced CARM1 distal sites, DNA motif analysis revealed that estrogen response element (ERE) was the most significant enriched motif embedded (P=1E-218), suggesting that CARM1 was recruited to ERα-bound enhancers in the presence of estrogen (**Figure 2E**). Indeed, around 84% of estrogen-induced CARM1 distal sites were found to have ERα binding (**Figure 2F**). In addition, binding motifs for Forkhead box proteins and AP-1/2 transcription factor subunit were highly enriched. To further support CARM1 co-localization with ERα, ERα binding was highly enriched on these estrogen-induced CARM1 distal sites shown by heat map and tag density plot (**Figure 2G**, the fourth column, and **Figure S2B**, the top panel on the right). More importantly, ERα binding was the most robust on these estrogen-induced CARM1 distal sites (**Figure S2B**, compare the right top panel to the left), which highly resembled the group of active enhancers reported recently that were essential for the cognate, estrogen-induced coding gene transcriptional activation 49. Enhancer characteristics, namely highly enriched H3K4me1/2, but low levels of H3K4me3, were evident from heat map and tag density plot analysis on those estrogen-induced CARM1 distal sites (**Figure 2G**, the fifth to the tenth columns, and **Figure S2B**, the second to the fourth panels, both left and right). Furthermore, these CARM1-bound enhancers were decorated with H3K27Ac histone marker and co-activator protein p300 and MED1 (**Figure 2G**, the eleventh to the sixteenth columns, and **Figure S2B**, the fifth and seventh panels, both left and right), but were devoid of repressive histone markers including H3K9me3 or H3K27me3 (**Figure 2G**, the seventeenth to twentieth columns, and **Figure S2B**, the ninth and tenth panels, both left and right). Occupancy of CARM1, ERα, H3K4me1/2/3, H3K27Ac, p300, MED1, H3K9me3 and H3K27me3 on representative active enhancers were shown, such as the ones in vicinity of estrogen-induced coding genes, *GREB1*, *TSKU*, *NRIP1*, *PGR*, *SIAH2* and* APOA1*(**Figure 2H**, **2I** and **Figure S2C**). We then integrated estrogen-induced CARM1 enhancer sites with estrogen-induced, CARM1-dependent genes, and found that around 85% of estrogen-induced, CARM1-dependent genes had CARM1 sites nearby, suggesting that CARM1 binding was generally required for the majority of estrogen-induced, CARM1-dependent gene expression. Taken together, our data suggested that CARM1 binds to ERα-bound active enhancers upon estrogen treatment, leading to the transcriptional activation of enhancers and cognate genes.

### Global mapping of CARM1 substrates reveals a large cohort of proteins with implications in estrogen receptor-mediated gene transcriptional activation

**Figure 3:**
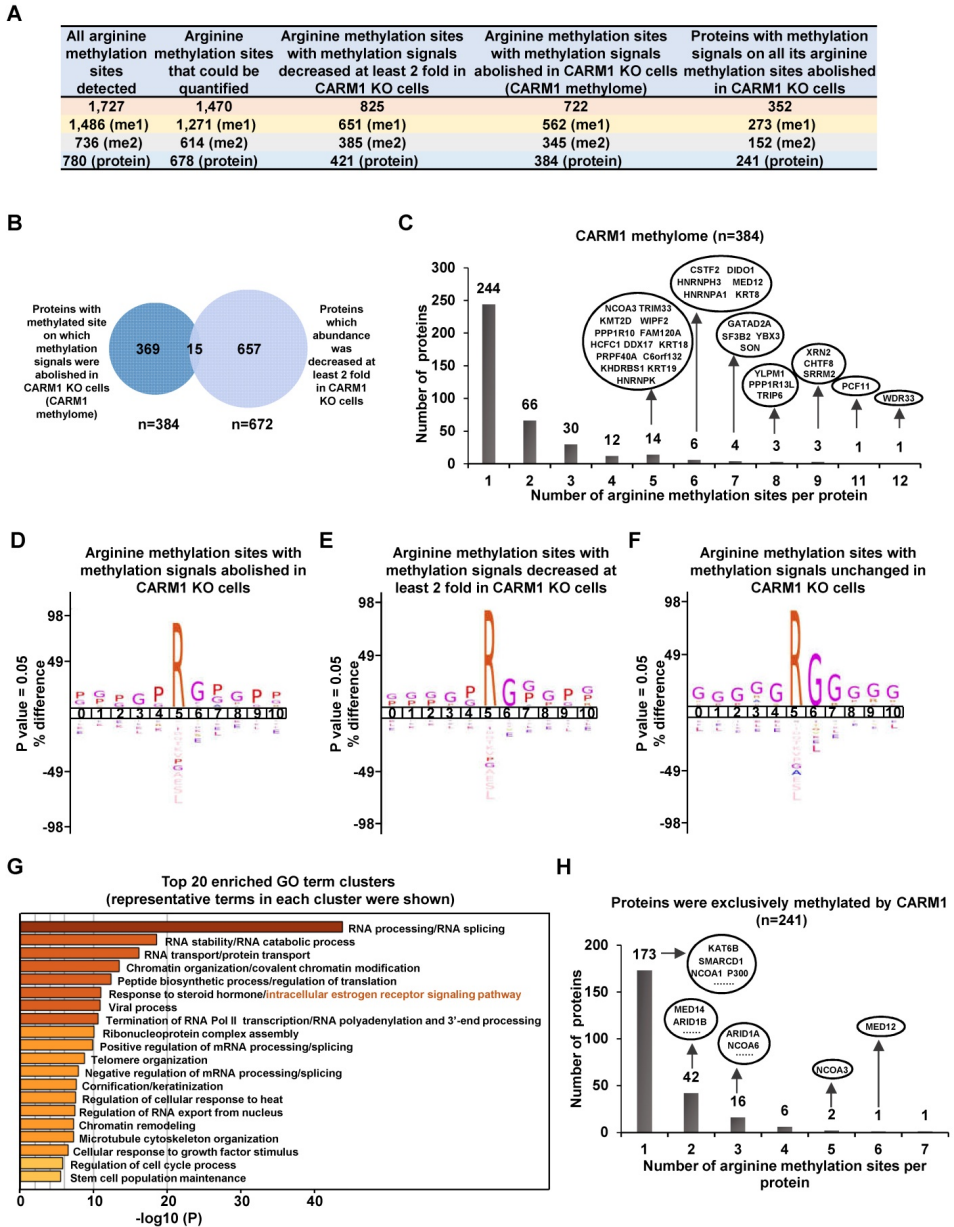
** Global mapping of CARM1 substrates.** (A) Table showing the number of (the sum of mono- and di-methylation sites after removing duplicates) all arginine methylation sites detected (column 1), arginine methylation sites could be quantified (column 2), arginine methylation sites with methylation signals decreased at least two-fold (column 3) or abolished (column 4) in CARM1 knockout (KO) cells detected from mass spectrometry analysis. The number of proteins encompass all these methylation sites was also shown (bottom lane). Proteins with at least one methylation site on which methylation signal was abolished in CARM1 KO cells were referred as “CARM1 methylome”. (B) Overlap between proteins in CARM1 methylome and proteins which abundance was decreased at least two fold in CARM1 KO cells. (C) The distribution of proteins in CARM1 methylome with different number of arginine methylation sites. All the proteins with more than four methylation sites were shown in oval. (D-F) Motif analysis using iceLogo for arginine methylation sites with methylation signals abolished (D), decreased at least two-fold (E) and unchanged (F) in CARM1 KO compared to control cells. (G) Gene ontology (GO) analysis using Metascape for CARM1 methylome. Representative terms from the top 20 enriched GO term clusters were shown. GO term “intracellular estrogen receptor signaling pathway” was highlighted in orange. (H) The distribution of proteins (exclusively methylated by CARM1) with different number of arginine methylation sites. Representative examples with implications in estrogen receptor-mediated transcriptional control were shown in oval.

CARM1 is a type I arginine methyltransferase, which can catalyze both mono- and asymmetrical di-methylation on arginine residues. To further explore the molecular mechanisms underlying CARM1 regulation of estrogen-induced gene transcriptional activation, we sought to globally map its methylation substrates by high-resolution mass spectrometry analysis of enriched peptides from anti-monomethyl- and anti-asymmetric dimethyl-arginine antibodies in SILAC labeled wild-type and CARM1 knockout cells (**Figure S3A**). It should be noted that these antibodies have been extensively validated and used in other studies to map methylated arginine sites in the proteome 8-12, 50. By using these antibodies, we have detected 1,727 methylated arginine sites in total, among which 1,486 sites have mono-methylation, 736 sites have di-methylation and 495 sites have both mono- and di-methylation (**Figure 3A**, the first column and**Table S1**). These 1,727 methylation sites were originated from 780 proteins, suggesting that there were often multiple methylated arginines in these proteins. Indeed, proteins, such as *TAF15*, *CHTF8*, *RBMX*, *WDR33*, *KRT18*, *TRIP6*, *PCF11*, *YLPM1*, *HNRNPA1*, *HNRNPK*, *XRN2*, *DIDO1*, *KRT8*, *RBMXL1* and *SRRM2*, have more than 10 arginine residues been methylated (**Figure S3B** and**Table S1**). Out of these 1,727 arginine methylation sites identified, 1,470 could be accurately quantified in wild-type versus CARM1 knockout cells (**Figure 3A**, the second column). The methylation signals on more than 56% of these methylation sites (825 out of 1,470) were found to be decreased at least two-fold in CARM1 knockout cells (**Figure 3A**, the third column and**Table S1**). Furthermore, methylation signals on 722 sites were completely abolished in CARM1 knockout cells, while the levels of proteins encompassing these sites barely changed, which we called as the bona fide substrates for CARM1(**Figure 3A**, the fourth column and** Figure 3B**). These 722 sites were originated from 384 proteins, and we named this large cohort of proteins as “CARM1-methylome” (**Figure 3A**, the fourth column and**Table S1**). To our best knowledge, this was the largest number of CARM1 substrates identified so far, which covered most of the known substrates characterized 9, 15, 51. Intriguingly, for CARM1 substrates with multiple arginine methylation sites, those sites often tended to form a cluster. For instance, for those CARM1 substrates with more than four arginine methylation sites identified, this type of cluster, without exception, could be observed, exhibiting a hypermethylation status (**Figure 3C, Figure S3C** and**Table S1**), which was similar as DNA hypermethylation 52, 53. Motif analysis for sequences in vicinity of those methylation sites on which methylation signals were completely abolished (n=722) or decreased at least two fold (n=825) revealed the proline-containing motifs, which was consistent with previous studies 9, 50 (**Figure 3D** and** 3E**). As expected, the well-characterized glycine and arginine-rich (GAR) motifs, which are typically methylated by other PRMTs, were detected for methylation sites whose abundance was unaffected in CARM1 knockout cells (**Figure 3F**). According to COSMIC (the Catalogue of Somatic Mutations in Cancer), mutation frequency at and in the proximity of CARM1-dependent arginine methylation sites (n=722) was significantly higher compared to that at all arginine sites in the proteome, indicating that CARM1 methylation sites as well as sequences in vicinity are vulnerable to mutations in cancers (**Figure S3D** and **S3E**). Gene ontology (GO) analysis for proteins in “CARM1 methylome” (n=384) revealed that they were implicated in diverse biological processes, such as those related to RNA biology (RNA splicing, RNA stability, RNA transport, RNA polyadenylation, RNA 3'-end processing etc.), chromatin organization/modification, chromatin remodeling, chromosome organization, protein translation, RNA Pol II-mediated transcription, viral process, telomere organization, keratinization, cellular response to heat/growth factor stimulus, microtubule cytoskeleton organization, regulation of cell cycle process and stem cell population maintenance (**Figure 3G**). Particularly, one of the most enriched clusters was implicated in intracellular estrogen receptor signaling pathway, which included proteins known to bind with chromatin to regulate gene transcription, such as MED12/14, NCOA1/3/6, KMT2C/2D, ARID1A/1B, SETD1A, P300/400 and several components in the SWI/SNF complex, supporting the notion that CARM1 might regulate estrogen/ ERα-mediated gene transcriptional activation through targeting these proteins (**Figure 3G**). Further examination of the “CARM1 methylome” revealed that 241 out of 352 proteins were exclusively methylated by CARM1 (i.e. methylation on all the arginine methylation sites found in a given protein was completely abolished in CARM knockout cells.) (**Figure 3A**, the fifth column). Proteins involved in intracellular estrogen receptor signaling pathway, such as MED12/14, NCOA1/3/6, ARID1A/1B, P300 and SMARCD1, were among the list of proteins exclusively methylated by CARM1 (**Figure 3H**).

It should be noted that some of the arginine methylation sites might escape from our analysis due to incomplete coverage of a given protein (*vide infra*). Taken together, identification of a large cohort of CARM1 substrates with implications in a variety of biological processes which are often dysregulated in cancer, particularly in intracellular estrogen receptor signaling pathway, and the hypermethylation status observed on these proteins underscores the significance of CARM1-mediated arginine methylation in breast carcinogenesis.

### MED12 is hypermethylated by CARM1 at a cluster of arginine residues at its carboxyl-terminus

**Figure 4:**
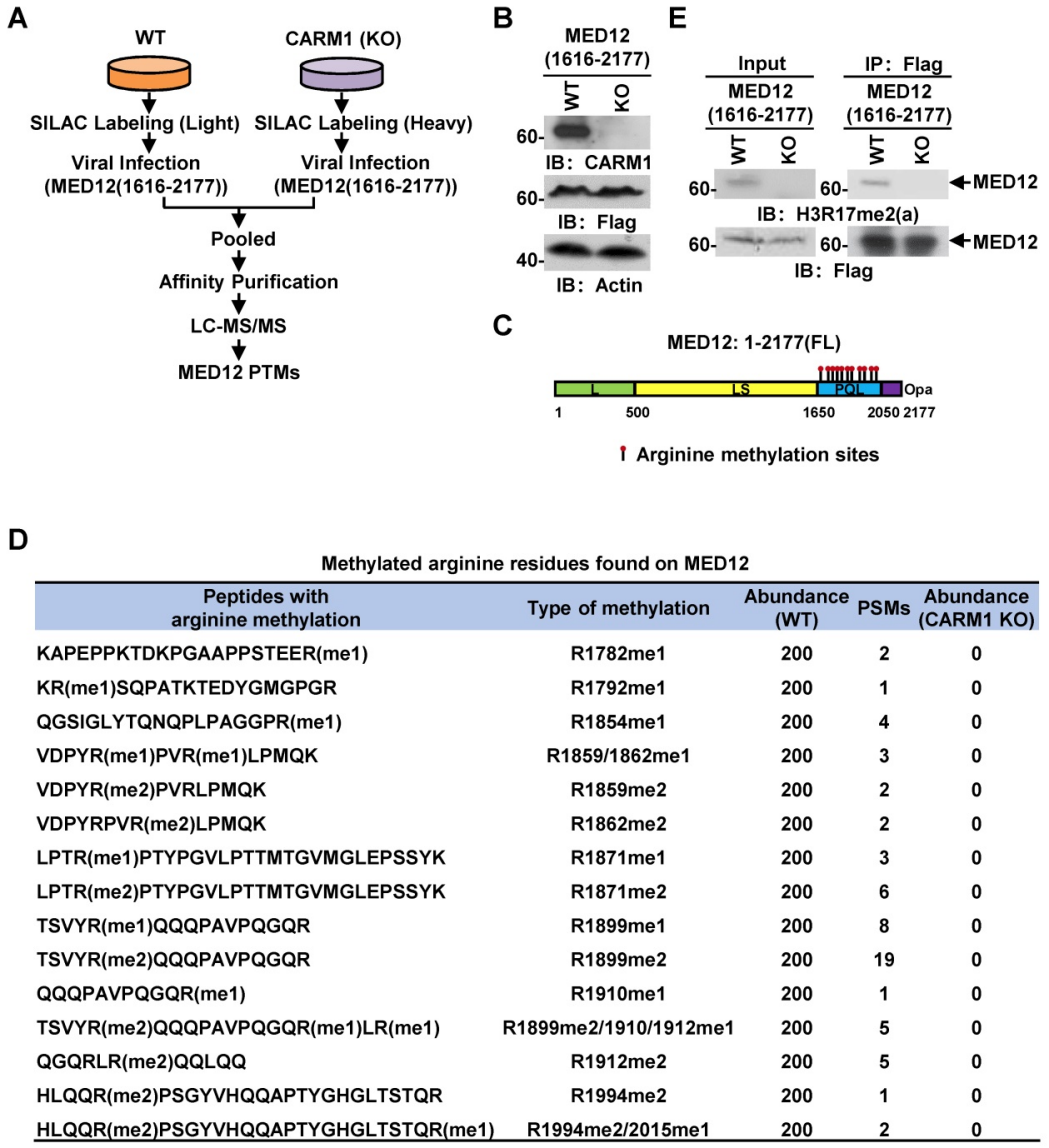
** MED12 is hypermethylated by CARM1.** (A) Experimental flowchart for detecting post translational modifications (PTMs) of C-terminus of MED12 (1616-2177) in wild type (WT) or CARM1 knockout (KO) MCF7 cells. (B) Cell lysates as described in (A) were subjected to immunoblotting (IB) analysis with antibodies as indicated. Actin was served as a loading control. (C) Schematic representation of the domain architecture of MED12 protein. Leucine-rich (L) domain (light green); Leucine-serine-rich (LS) domain (yellow); Proline-glutamine-leucine (PQL) domain (light blue); Poly-glutamine (Opa) domain (purple). Arginine methylation sites identified in the PQL domain were shown by matchsticks. (D) Methylated arginine residues identified in the C-terminus of MED12 following the protocol as described in (A). me1: mono-methylation; me2: di-methylation. PSM: peptide spectrum match. (E) WT and CARM1 KO cells were infected with lenti-viral vectors expressing Flag-tagged MED12 C-terminus (1616-2177), lysed and subjected to immunoprecipitation using anti-Flag antibody followed by immunoblotting (IB) analysis with antibodies as indicated. Anti-H3R17me2(a) antibody was used to detect methylated MED12.

Out of the many substrates identified, we focused on MED12, a component in the mediator complex, to study the function of CARM1 methylation, as our previous study demonstrated that it interacts with CARM1 and is involved in estrogen-induced transcriptional activation. However, the full spectrum of arginine methylation sites on MED12 and the function of such methylation remain to be characterized 54. From our MS analysis above, MED12 was exclusively methylated by CARM1 at multiple arginine residues at its carboxyl (C)-terminus, which was consistent with our previous observations that CARM1 interacts with and only methylates the C-terminus of MED12 54. To uncover the full spectrum of arginine methylation sites in MED12 by CARM1 in cells, wild-type or CARM1 knockout cells were subjected to SILAC labeling, infected with Flag-tagged C-terminus of MED12, pooled and followed by affinity purification and mass spectrometry (MS) analysis (**Figure 4A** and** 4B**). MED12 was found to be heavily methylated at multiple arginine sites, including arginine 1782 mono-methylation (R1782me1), R1792me1, R1854me1, R1859me1/2, R1862me1/2, R1871me1/2, R1899me1/2, R1910me1, R1912me1/2, R1994me2 and R2015me1 (**Figure 4C** and** 4D**, **Figure S4A** and**Table S2**). Importantly, the methylation on all sites was abolished in CARM1 knockout cells (**Figure 4D**), further supporting MED12 C-terminus was heavily and exclusively methylated by CARM1. To our best knowledge, the arginine methylation sites we identified for MED12 here not only covered all the reported ones in the literature but also revealed a number of new ones, highlighting the hypermethylation strategy utilized by CARM1 on specific substrates. Abolishment of arginine methylation on MED12 in CARM1 knockout cells was confirmed when immunoprecipitated MED12 was subjected to immunoblotting using anti-H3R17me2 (a) antibody, which could largely recognize the methylated substrates for CARM1 (**Figure 4E,** top panels). MED12 was equally pulled down in both wild-type and CARM1 knockout cells (**Figure 4E**, bottom panels). Taken together, exemplified by MED12, our data suggested that the substrates we identified are bona fide CARM1 substrates.

### CARM1-mediated MED12 methylation is involved in estrogen-induced gene transcriptional activation

**Figure 5:**
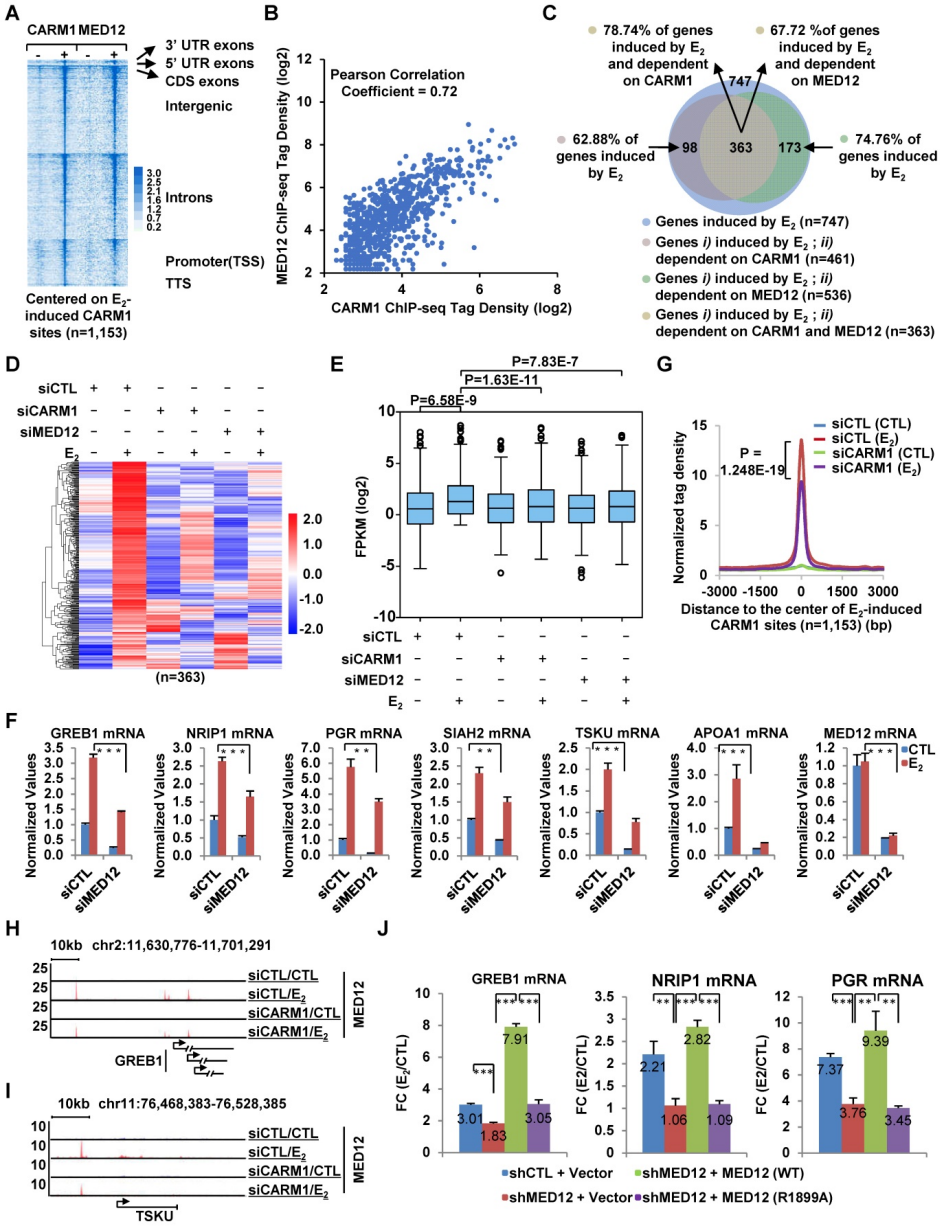
** CARM1-mediated MED12 methylation is involved in estrogen-induced gene transcriptional activation.** (A) Heat map representation of CARM1 and MED12 ChIP-seq tag density centered on estrogen-induced CARM1 sites (± 3,000 bp). (B) Correlation between the ChIP-seq tag density (log2) of CARM1 and MED12 on estrogen-induced CARM1 sites. (C) MCF7 cells were transfected with siCTL, siCARM1 or siMED12, and treated with or without estrogen (E_2_, 10^-7^ M, 6 hrs) followed by RNA-seq analysis. Estrogen-induced genes which were dependent on both CARM1 and MED12 were shown by Pie chart. (D, E) Heat map (D) and box plot (E) representation of the expression levels (FPKM) for genes induced by estrogen and dependent on both CARM1 and MED12 as described in (C). Heat map: z-score normalized FPKM; box plot: FPKM (log2). (F) MCF7 cells were transfected with siCTL or siMED12 in stripping medium for three days, and treated with or without estrogen (E_2_, 10^-7^ M, 6 hrs), followed by RNA extraction and RT-qPCR analysis to examine the expression of selected estrogen-induced coding genes as indicated (± s.e.m., **P<0.01, ***P<0.001). (G) MCF7 cells were transfected with siCTL or siCARM1 in stripping medium for three days, and treated with or without estrogen (E_2_, 10^-7^ M, 1 hr) followed by ChIP-seq with anti-MED12 antibody. MED12 ChIP-seq tag density distribution centered on estrogen-induced CARM1 sites was shown (± 3,000 bp). (H, I) The binding of MED12 as described in (G) was shown for specific genes, as indicated. (J) MCF7 cells were infected with lenti-viral vectors expressing shRNA targeting MED12 together with or without Flag-tagged wild type (WT) MED12 or MED12 mutants with arginine 1899 replaced by alanine (R1899A), and then treated with or without estrogen (E_2_, 10^-7^ M, 6 hrs) followed by RT-qPCR analysis to examine the expression of selected estrogen-induced genes as indicated (± s.e.m., **P<0.01, ***P<0.001). Data was presented as fold induction by estrogen.

We next ask whether CARM1-mediated MED12 methylation is important for estrogen-induced gene transcriptional activation. Firstly, both heat map (**Figure 5A**) and tag density plot (**Figure S2B**, the eighth panel on the right) demonstrated that MED12 was recruited to estrogen-induced CARM1 binding sites, and ChIP-seq tag counts of MED12 and CARM1 were highly correlated on estrogen-induced CARM1 sites (**Figure 5B**). Secondly, MED12 and CARM1 exerted similar effects on estrogen-induced gene transcriptional activation based on RNA-seq analysis (**Figure 5C**). The expression of around 75% and 63% of estrogen-induced genes were attenuated following MED12 and CARM1 knock-down, respectively, with the vast majority of MED12 and CARM1-affected genes overlapped (**Figure 5C**). The impact of MED12 and CARM1 on the expression of those commonly regulated genes was shown by heat map (**Figure 5D**), and statistical test was performed (**Figure 5E**). Effects of MED12 on representative estrogen-induced target genes were confirmed by RT-qPCR analysis (**Figure 5F**). MED12 knockdown appeared to have no significant impact on the expression of CARM1 (**Figure S5A** and **S5B**). Our RNA-seq experiments were highly reproducible between two independent siRNAs targeting MED12 or CARM1 (**Figure S5C** and**Figure S1G**).

Having demonstrated that MED12 is also co-recruited to CARM1-bound ERα active enhancers, and regulates estrogen-induced gene transcriptional activation, we then asked whether CARM1-mediated methylation regulates MED12 binding on CARM/ERα-bound active enhancers. As shown by tag density plot, knockdown of CARM1 significantly attenuated the binding of MED12 on estrogen-induced CARM1 binding sites (**Figure 5G**). MED12 binding in response to CARM1 knockdown was shown on representative estrogen-induced gene promoter as well as cognate enhancer regions (**Figure 5H** and **5I**). The impact of CARM1 on MED12 binding was well correlated between two independent siRNAs targeting CARM1 (**Figure S5D**). The mRNA and protein level of MED12 was unchanged when knocking down of CARM1, excluding the possibility that CARM1 regulation of MED12 binding on chromatin was due to its effects on MED12 expression (**Figure S5E** and **S5F**). CARM1 regulation of MED12 binding suggested that CARM1-mediated methylation might be involved in MED12 function in estrogen-induced gene transcriptional activation. To test this, MCF7 cells were infected with lenti-viral vector expressing shRNA targeting MED12 together with or without vectors expressing wild-type MED12 and MED12 mutants with substitution of each of the arginine sites identified above to alanine (R1782A, R1792A, R1854A, R1859A, R1862A, R1871A, R1899A, R1910A, R1912A, R1994A and R2015A), and then treated with or without estrogen followed by RT-qPCR analysis to examine the expression of selected estrogen-induced genes. It was found that shRNA-mediated knockdown of MED12 attenuated the estrogen-induced expression of these genes, which could be rescued by over-expressed MED12 (WT). However, none of the mutants, except MED12 (R1899A), exhibited a consistent effect in terms of their ability to rescuing gene transcription, with some genes could be rescued but not others (data not shown).

MED12 (R1899A) failed to rescue the estrogen-induced expression of any of the genes examined, highlighting the importance of this site, which was consistent with the observation that the number of PSMs (peptide spectrum match) for R1899 methylation detected in our MS analysis was the most among all methylation sites (**Figure 4D**, **Figure 5J** and **Figure S5G**). Both MED12 (WT) and MED12 (R1899A) appeared to have no significant impact on the expression of CARM1 (**Figure S5H**). The knockdown efficiency of shRNA targeting MED12 and the expression of MED12 (WT) and MED12 (R1899A) were shown (**Figure S5I**). Taken together, CARM1-mediated methylation on MED12 is required for MED12 function in estrogen-induced gene transcriptional activation.

### Arginine methylation reader protein TDRD3 recognizes CARM1-mediated MED12 methylation and is involved in estrogen-induced gene transcriptional activation

**Figure 6:**
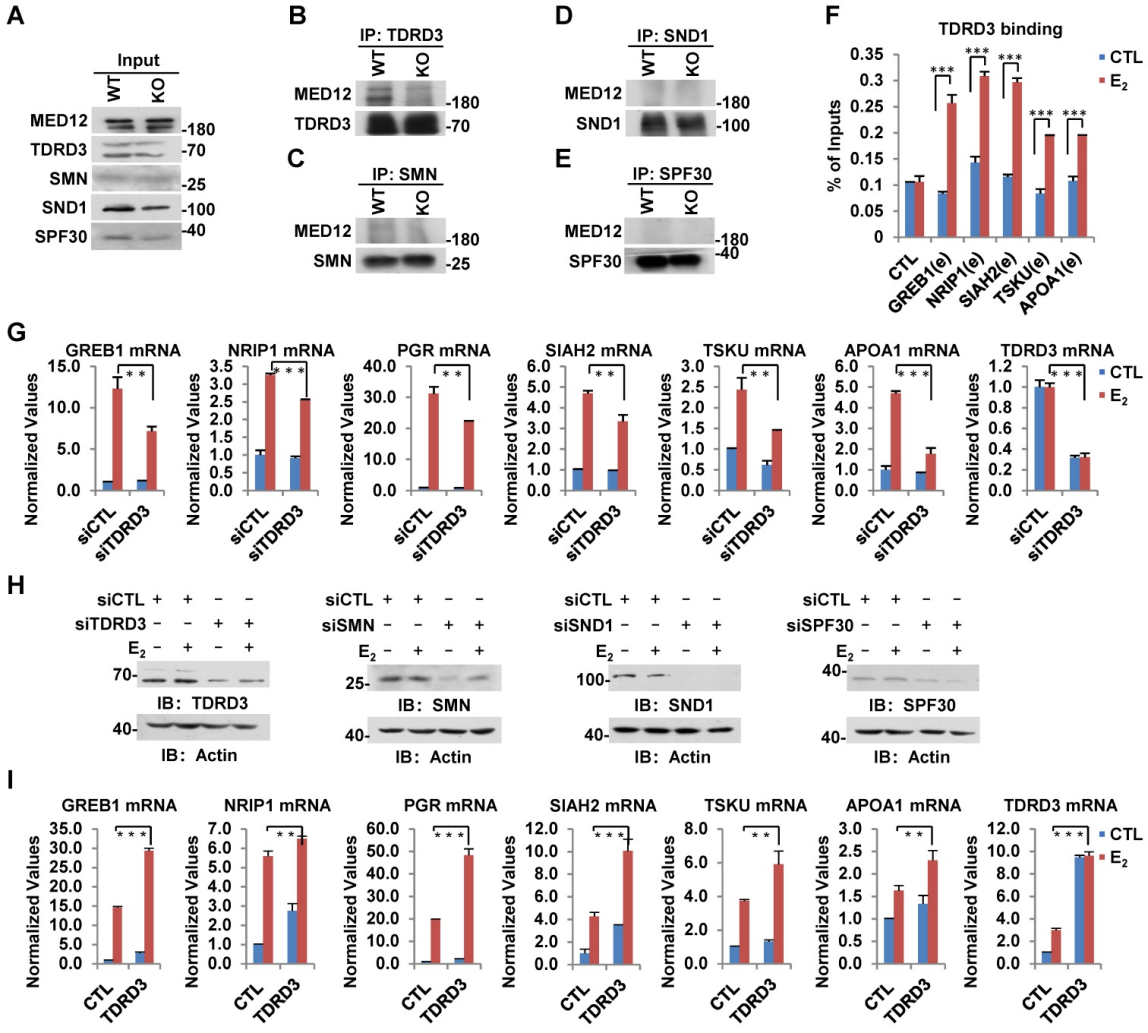
** TDRD3 reads MED12 methylation and is involved in estrogen-induced gene transcriptional activation.** (A-E) Wild type (WT) and CARM1 KO MCF7 cells treated with estrogen were subjected to immunoprecipitation (IP) with anti-TDRD3 (B), SMN (C), SND1 (D) or SPF30 (E) antibody followed by immunoblotting (IB) with antibodies as indicated. Input was also shown (A). (F) MCF7 cells treated with or without estrogen (E_2_, 10^-7^ M, 1 hr) were subjected to ChIP with anti-TDRD3 antibody followed by qPCR analysis with primers specifically targeting enhancer (e) regions as indicated. A control (CTL) region was also included. ChIP signals were presented as percentage of inputs (± s.e.m., ***P<0.001). (G, I) MCF7 cells were transfected with siCTL, siTDRD3, siSMN, siSND1 or siSPF30 (G), or infected with lenti-viral vectors expressing TDRD3, SMN, SND1 or SPF30 (I) in stripping medium for three days, and treated with or without estrogen (E_2_, 10^-7^ M, 6 hrs), followed by RNA extraction and RT-qPCR analysis to examine the expression of selected estrogen-induced genes as indicated (± s.e.m., **P<0.01, ***P<0.001). Data for siSMN, siSND1, siSPF30, SMN, SND1 and SPF30 were shown in Figure S6A, S6B, S6C, S6D, S6E and S6F, respectively. (H) MCF7 cells as described in (G) were subjected to immunoblotting analysis with antibodies as indicated. Actin was served as a loading control.

In humans, several tudor-domain-containing proteins, including TDRD3, SMN, SND1 and SPF30, have been shown to read CARM1-mediated asymmetric dimethylation on arginine, through which methylation exerts its function in gene transcriptional control as well as other biological processes 47. To further explore how CARM1-mediated methylation on MED12 regulates estrogen-induced gene transcriptional activation, we sought to look for the reader protein which can recognize arginine methylation on MED12. Wild-type and CARM1 knockout cells were subjected to IP with antibodies specific for TDRD3, SMN, SND1 or SPF30 and followed by immunoblotting analysis with anti-MED12 antibody. It was found that MED12 specifically interacted with TDRD3, but not SMN, SND1 or SPF30 in WT cells (**Figure 6B-E**, left lane in upper panel). More importantly, the interaction between MED12 and TDRD3 was abolished in CARM1 KO cells, suggesting CARM1, presumably CARM1-mediated methylation, is critical for their interaction (**Figure 6B**, right lane in upper panel). The loading of and immunoprecipitated TDRD3, SMN, SND1 and SPF30 in WT and CARM1 KO cells was comparable (**Figure 6A** and **Figure 6B-6E**, bottom panels). TDRD3 was found to be recruited to CARM1/MED12-bound ERα active enhancers in response to estrogen treatment, consistent with the observation that it can interact with MED12 (**Figure 6F**). Furthermore, knockdown of TDRD3 led to a significant reduction of estrogen-induced gene transcriptional activation, such as *GREB1*, *NRIP1*, *PGR*, *SIAH2*, *TSKU* and *APOA1*, while knockdown of SMN, SND1 or SPF30 had no effects or exhibited inconsistent effects (**Figure 6G** and **Figure S6A-S6C**). The knockdown efficiency of siRNA targeting TDRD3, SMN, SND1 and SPF30 were examined by RT-qPCR as well as immunoblotting analysis (**Figure 6G**, **Figure S6A-S6C** and** Figure 6H**). As expected, overexpression of TDRD3, but not SMN, SND1 or SPF30, further increased the induction by estrogen (**Figure 6I** and **Figure S6D-S6F**). Taken together, CARM1-mediated methylation recruits transcriptional coactivator TDRD3 to ERα-bound active enhancer in response to estrogen treatment, leading to gene transcriptional activation.

### CARM1 is required for ERα-positive breast cancer cell growth and tumorigenesis

**Figure 7:**
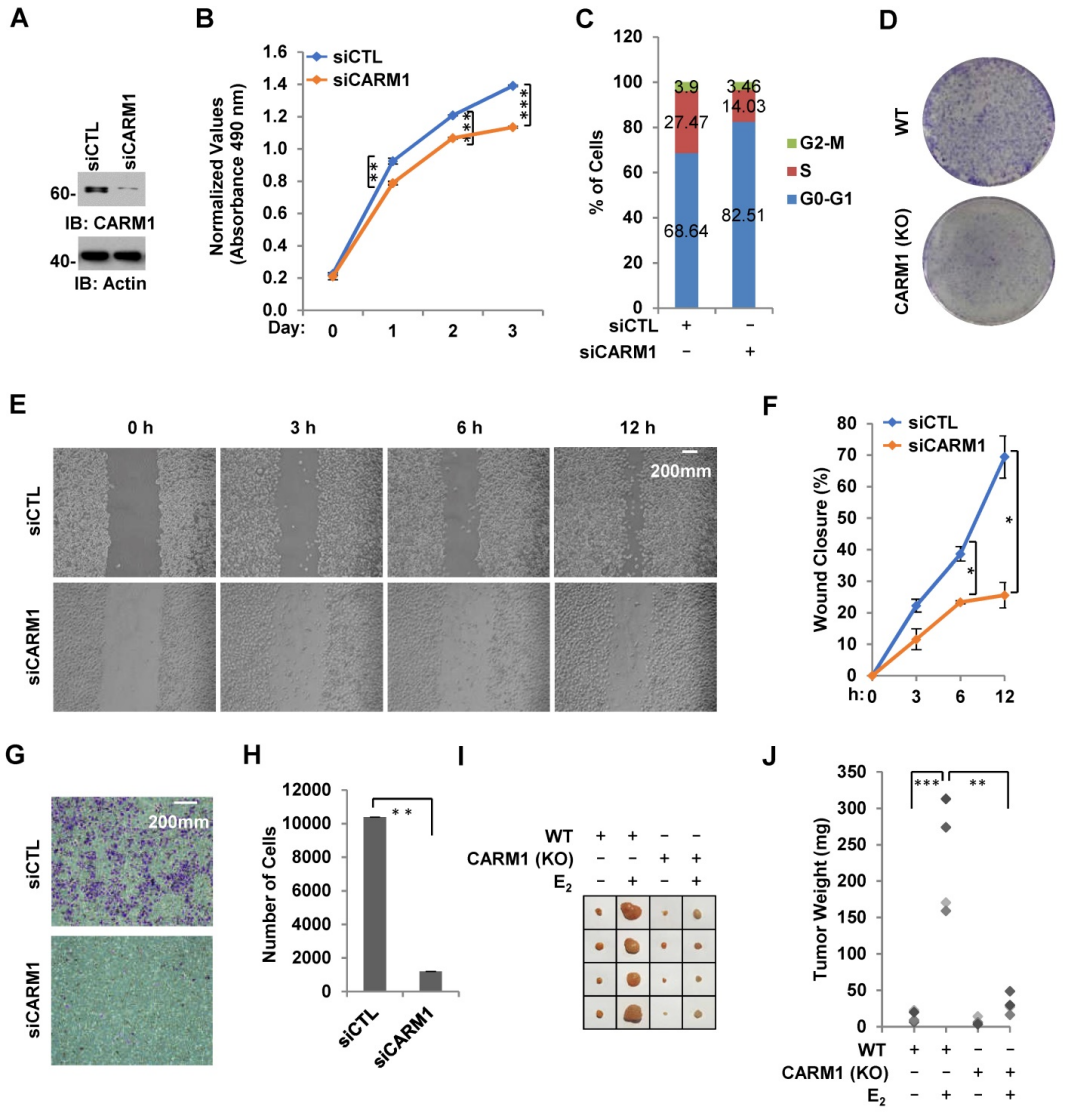
** CARM1 is required for estrogen-induced breast cancer cell growth and tumorigenesis.** (A) MCF7 cells transfected with siCTL or siCARM1 were subjected to immunoblotting using antibodies as indicated. (B, C) MCF7 cells as described in (A) were subjected to cell proliferation assay (B) and FACS analysis (C) (± s.e.m., **P<0.01, ***P<0.001). (D) Colony formation assay was performed in WT and CARM1 KO MCF7 cells. (E) MDA-MB-231 cells transfected with siCTL or siCARM1 for 48 hrs were both re-seeded at full confluence and then subjected to wound-healing assay. (F) Quantification of wound closure shown in (E) (± s.e.m., *P<0.05). (G) MDA-MB-231 cells as described in (E) were both re-seeded at the same confluence and then subjected to trans-well assay. (H) Quantification of (G) (± s.e.m., **P<0.01). (I) WT and CARM1 KO MCF7 cells were injected subcutaneously into female BALB/C nude mice for tumor xenograft experiments. (J) Tumor weight as shown in (I) (± s.e.m., *P<0.05, **P<0.01).

Due to its critical role in estrogen/ERα-induced gene transcriptional activation, we tested whether CARM1 regulates ERα-positive breast cancer cell growth and tumorigenesis. Using MCF7 breast cancer cell line as a model system, we demonstrated that cell proliferation rate was decreased significantly and cells were arrested in G1 phase when CARM1 was knocked down (**Figure 7A-C**). Similarly, CARM1 KO MCF7 cells also exhibited slower proliferation rate and more cells at G1 phase (**Figure S7A** and **S7B**). The effects of CARM1 on MCF7 cell proliferation was further demonstrated by colony formation assay (**Figure 7D**). Requirement of CARM1 for cell proliferation was also observed in two other ERα-positive breast cancer cell lines (**Figure S7C-S7F**). We also found that CARM1 knockdown decreased cell migration analyzed by wound healing assay and transwell assay (**Figure 7E**-**7H**). To test CARM1 effects on tumor growth *in vivo*, we injected nude mice subcutaneously with control or CARM1 knockout MCF7 cells, and then treated with or without estrogen. Exogenous administration of estrogen was to sustain the growth of tumor in mice. Indeed, tumor volume was dramatically induced when mice were estrogen-treated compared to the control group after four weeks. Importantly, CARM1 knockout significantly attenuated the effects of estrogen-induced tumorigenesis (**Figure 7I-7J**). Taken together, our data suggested that CARM1 is required for ERα-positive breast cancer cell growth and tumorigenesis, exemplified by MCF7 cells.

## Discussion

Members in the PRMT protein family, particularly PRMT4/CARM1, have been shown to be involved in the development of breast cancers 16, 17. However, the molecular mechanisms underlying CARM1 regulation of breast cancer development remain incompletely understood, which is partially due to the lack of genomic, transcriptomic and proteomic characterization of CARM1 chromatin-binding sites, transcriptional targets and methylation substrates in a systematic way. In the current study, utilizing MCF7 cells as a model system, we focused on investigating CARM1 function in ERα-positive breast cancers. It was found that CARM1 was specifically recruited to ERα-bound active enhancers, and regulated cognate gene transcriptional activation in the presence of estrogen. Furthermore, global mapping of CARM1 substrates revealed that a large cohort of proteins with functions in a myriad of biological processes, including intracellular estrogen receptor signaling pathway, were methylated by CARM1. A large number of these proteins were exclusively methylated by CARM1 on array of clustered arginine sites, exhibiting a hypermethylation status. Exemplified by MED12, CARM1-mediated methylation was shown to be critical for estrogen/ERα-induced gene transcriptional activation. In consistent with the observation that it plays a prominent role in estrogen/ERα-induced gene transcriptional activation, CARM1 was demonstrated to be required for ERα-positive breast cancer cell growth and tumorigenesis.

Through ChIP-seq, we demonstrated that CARM1 was specifically and robustly recruited onto a group of ERα-bound enhancers, which were reported to be critical for the transcriptional activation of cognate estrogen-induced target genes 49. Indeed, knockdown of CARM1 attenuated the transcriptional activation of vast majority of estrogen-induced genes examined by RNA-seq analysis. Besides its direct role in activating estrogen-induced transcription through binding on ERα-occupied enhancers, CARM1 was noticed to affect estrogen-induced ERα degradation (Figure S1H), which might, at least partially, contribute to its function in estrogen-induced gene transcriptional activation. To further explore the molecular mechanisms underlying CARM1 regulation of enhancer and cognate gene transcriptional activation, we systematically characterized the “CARM1 methylome” (substrates) by mass spectrometry analysis. To our surprise, nearly half of the identified arginine methylation, encompassed by 384 proteins, was CARM1-dependent, further expanding the number of CARM1 substrates 9, 40, 55, 56. To our best knowledge, this is the largest number of CARM1 substrates identified so far, which might help to illustrate the underlying molecular mechanisms of CARM1 function in a wide range of biological processes, both physiology and pathology 33, 43, 55, 57-59. For instance, a large group of substrates were implicated in RNA alternative splicing, such as SRSF1/2/6/9, U2AF1, SF3B2 and several members in the HNRNP protein family, which is consistent with the reported function of CARM1 in RNA alternative splicing 60. Therefore, the contribution of CARM1-mediated methylation on these proteins to its function in gene alternative splicing warrants future investigation. To our particular interest, CARM1 was also found to methylate a group of proteins with implications in estrogen receptor-mediated signaling pathway, such as acetyltransferase (P300, P400, KAT6B, NCOA1 and NCOA3), lysine methyltransferase (KMT2C and KMT2D) and components in the SWI/SNF, NuRD and mediator complex. Out of the many CARM1 substrates with implications in estrogen receptor-mediated signaling pathway, we focused on MED12 to investigate the function of CARM1-catalyzed methylation. A cluster of arginine residues (R1782, R1792, R1854, R1859, R1862, R1871, R1899, R1910, R1912, R1994 and R2015) were found to be methylated at the C-terminus of MED12, which covered all the methylation sites currently reported in a comprehensive protein post-translational modification database (phosphosite.org). More importantly, the methylation on all these residues was completely abolished when CARM1 was knocked out, suggesting that MED12 was exclusively methylated by CARM1 and underscoring the significance of CARM1-mediated methylation. Interestingly, functional characterization revealed that mutation of each single methylation site, except R1899, appeared not to affect MED12 transcriptional activator activity consistently on selected estrogen target genes, suggesting that creation of this type of hypermethylation might ensure the recruitment of arginine methylation reader proteins, to activate gene transcription. Indeed, when we examined the other CARM1 methylation substrates identified in our MS analysis, hypermethylation appeared to be a common strategy utilized by CARM1 such that clustered methylation sites could be found in a large number of proteins. Proteins, such as WDR33, PCF11, XRN2, CHTF8, SRRM2, YLPM1, TRIP6, PPP1R13L, GATAD2A, SF3B2, YBX3, SON, CSTF2, DIDO1, HNRNPH3, HNRNPA1, MED12 and KRT8, had at least five arginine residues forming a cluster. We must emphasize that MED12 is just one of the many proteins being methylated by CARM1, and we propose that other proteins and the associated methylation, similar as MED12, might be as important as MED12 in terms of activating estrogen/estrogen receptor-induced gene transcription, which deserves a systematic screening to assure such as a function role. CARM1's dominant role in estrogen/estrogen receptor-induced gene transcriptional activation would be the combinatory effects of methylation imposed on many substrates similar as MED12. It should be noted that there were limitations in our approaches to identifiy CARM1 methylome. Only one single CARM1 knockout clone was used, and therefore further systematic validation would be required. In addition, due to the diversified arginine methylation motifs embedded in the proteome, some of the arginine-methylated proteins might escape from detection by the anti-monomethyl- and anti-asymmetric dimethyl-arginine antibodies used in this study.

**Figure 8:**
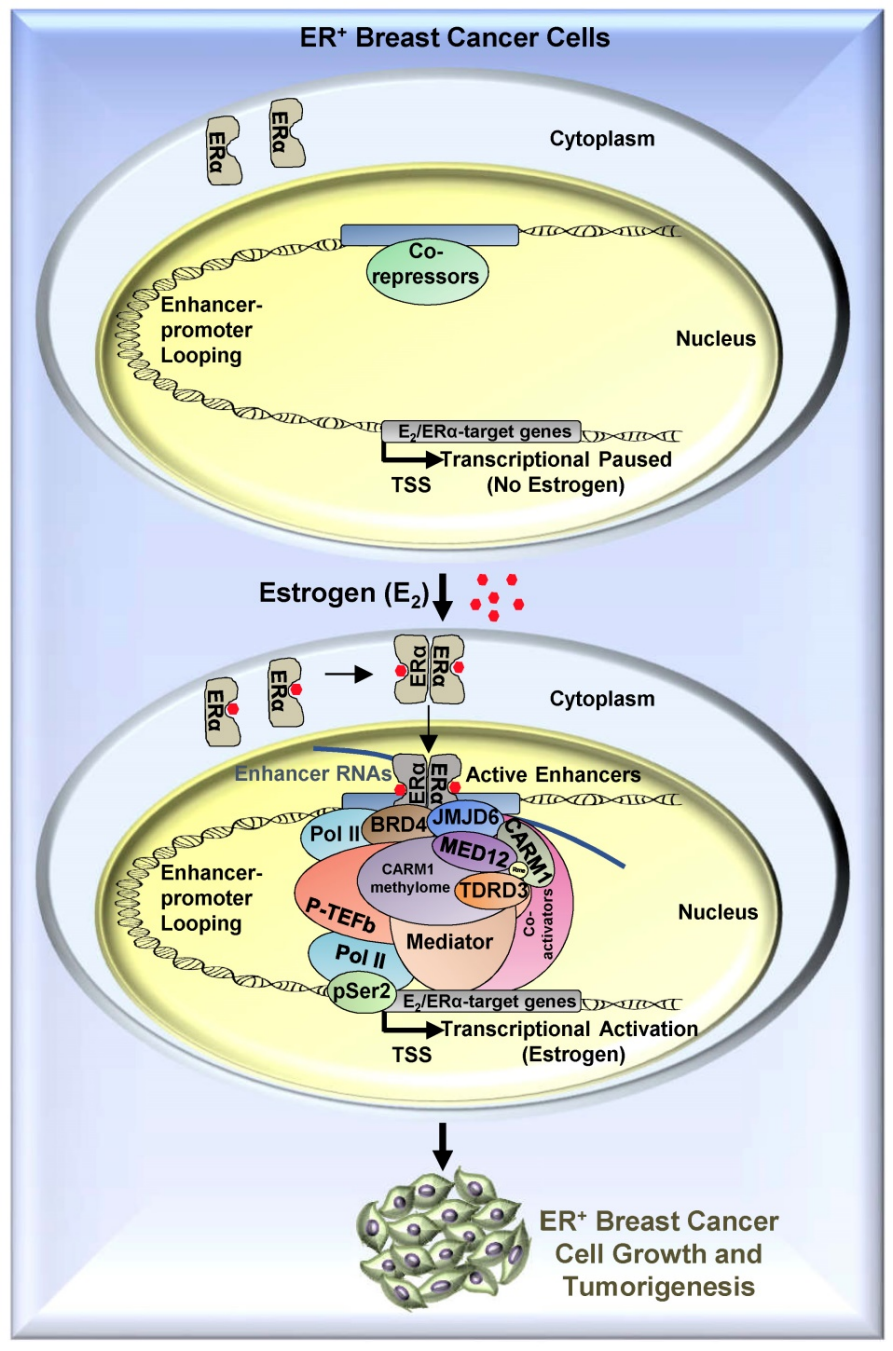
** A proposed model of CARM1 function in estrogen-induced gene transcriptional activation, breast cancer cell growth and tumorigenesis.** Based on the findings in this manuscript along with our previous report 54, we proposed that, upon estrogen stimulation, a coactivator complex constituting BRD4, JMJD6, CARM1, CARM1 substrates (represented by MED12), TDRD3 and others, was recruited to ERα-bound active enhancers, leading to the transcriptional activation of active enhancers as well as cognate estrogen/ERα-target genes. During the process of this gene activation event, CARM1 was found to hypermethylate a cohort of proteins, such as MED12, with implications in intracellular ERα-mediated signaling. CARM1-mediated methylation was critical for the recruitment of coactivator protein TDRD3 to activate estrogen/ERα-target genes. Prolonged exposure to high levels of estrogen will lead to the constitutive activation of this gene program, uncontrolled breast cancer cell growth and eventually breast cancer.

To search for the methylation reader which reads methylated MED12, we focused on tudor-domain containing protein family, in which TDRD3, SMN, SPF30 and SND1 have been shown to recognize asymmetrical di-methylarginines. It was found that TDRD3 specifically read MED12 methylation, which was consistent with a recent report showing that arginine 1899 (R1899) in MED12 was methylated and such methylation was recognized by TDRD3 40. To strengthen the observed interaction between TDRD3 and methylated MED12, TDRD3 was found to be recruited to CARM1 and MED12 co-bound ERα active enhancers, and to be essential for estrogen-induced gene transcriptional activation. Thus, to complement the molecular axis/network constituted by BRD4, JMJD6, CARM1 and MED12 we reported previously, a new player, TDRD3, was added, which together is critical for estrogen-induced transcriptional activation (**Figure 8**). In this axis, BRD4 served as the upstream protein to bring JMJD6 on, and JMJD6 was required for CARM1/MED12 to bind with chromatin, which further recognized by TDRD3.

Our data thereby revealed that CARM1 is required for estrogen/ERα-induced transcriptional activation, breast cancer cell growth and tumorigenesis, suggesting CARM1 might serve as a potential drug target in ERα-positive and endocrine therapy-resistant breast cancer. Particularly, CARM1 function in breast cancer is, at least partially, mediated through its methyltransferase activity targeting a large number of proteins. Therefore, developing small molecule inhibitors targeting CARM1 enzymatic activity or peptides mimicking sequence motifs in CARM1 methylation sites will provide an additional therapeutic adjunct for ERα-positive and endocrine therapy-resistance breast cancers.

## Methods and Materials

### Cloning Procedures

Lenti-viral vectors expressing full length (FL) or C-terminus (aa1616-2177) of MED12 was described previously 54. MED12 point mutations were generated by over-extension PCR method using Transstart fastpfu fly polymerase (TransGen Biotech). TDRD3, SMN, SND1 and SPF30 were PCR-amplified from cDNAs by using Transstart fastpfu fly polymerase and then cloned into pBoBi expression vector. 3XFlag- and 3XHA-tag were added to the amino- and carboxy-terminus of TDRD3, SMN, SND1 and SPF30, respectively, when cloned into pBoBi vector. ShRNA targeting CARM1 or MED12 was cloned into lenti-viral pLKO.1 vector with AgeI and EcoRI (targeting sequence: GATAGAAATCCCATTCAAA (CARM1); CAGCAATCTCTGAGACCAA (MED12)).

### SiRNA and Plasmids Transfection, Lenti-viral Vectors Packaging and Infection

Transfection of siRNA targeting CARM1 (5'-GAUAGAAAUCCCAUUCAAA-3' and 5'-GUAACCUCCUGGAUCUGAA-3'), MED12 (5'-GUACUUAGAUGAUUGCAAA-3' and 5'-UCACUCAUCUCAUGUUAUA-3'), TDRD3 (5'-GCAGUGGAUUACCUAGAAA-3'), SMN (5'-GCAGUGGAUUACCUAGAAA-3'), SND1 (5'-AAGGAAACTTGCCTTATCA-3') and SPF30 (5'-GGAGGACAGTGGCAACAAA-3') (RiboBio Co., Ltd.) were performed using Lipofectamine 2000 (Invitrogen) according to the manufacturer's protocol. Plasmid transfections were performed using Polyethyleneimine (PEI, Polysciences) according to the manufacturer's protocol.

Lenti-viral vectors packaging and infection: HEK293T cells were seeded in culture plates coated with poly-D-lysine (0.1% (w/v), Sigma, P7280) and transfected with lenti-viral vectors together with packaging vectors, pMDL, VSVG and REV, at a ratio of 10:5:3:2 using Polyethyleneimine (PEI, Polysciences) for 48 hrs according to the manufacturer's protocol. Virus was collected, filtered and added to MCF7 cells in the presence of 10 μg/mL polybrene (Sigma, H9268), followed by centrifugation for 30 mins at 1,500 g at 37 °C.

### RNA Isolation and RT-qPCR

RNA Isolation and RT-qPCR was performed as described previously 54. RNA samples from three biological repeats were pooled together for RT-qPCR analysis, and at least three technical repeats have been done for each pooled sample. Standard error of the mean is depicted. Sequence information for all primers used to check gene expression was presented in Table S3.

### Immunoblotting and Immunoprecipitation

Protein immunoprecipitation and immunoblotting were performed as described previously 54.

### Cell Proliferation Assay, FACS (Fluorescence-activated cell sorting) Analysis, Colony Formation Assay, Wound Healing Assay, Trans-well Assay and Tumor Xenograft Assay

Cell viability was measured by using a CellTiter 96 AQueous one solution cell proliferation assay kit (Promega) following the manufacturer's protocol. Briefly, MCF7, T47D and BT474 cells were transfected with control siRNA (siCTL) or siRNA specifically targeting CARM1 (siCARM1), and maintained in normal growth medium for different time points followed by cell proliferation assay. When WT and CARM1 (KO) MCF7 cells were subjected to cell proliferation assay, cells were seeded at the same density and maintained in normal growth medium for different time points followed by cell proliferation assay. To measure cell viability, 20 μl of CellTiter 96 AQueous one solution reagent was added per 100 μl of culture medium, and the culture plates were incubated for 1 hr at 37 ℃ in a humidified, 5% CO_2_ atmosphere incubator. The reaction was stopped by adding 25 μl of 10% SDS. Data was recorded at wavelength 490 nm using a Thermo Multiskan MK3 Microplate Reader.

For FACS analysis, cells were trypsinized, washed with PBS and fixed with ethanol at 4^o^C overnight. Cells were then washed with PBS and stained with PI/Triton X-100 staining solution (0.1% (v/v) Triton X-100, 0.2mg/mL DNase-free RNase A (Sigma), 0.02mg/mL propidium iodide (Roche)) at 37^o^C for 15 mins. DNA content was then measured and about 10^5^ events were analyzed for each sample. Data were analzsed using ModFit LT (Verity Software House).

For colony formation assays, 2,000 cells, infected with control shRNA (shCTL) or shRNA specifically targeting CARM1 (shCARM1), were maintained in a 6-well plate, and colonies were examined 10 days after. Briefly, colonies were fixed with methanol/acid solution (3:1) for 5 mins and stained with 0.1% crystal violet for 15 mins.

For wound-healing assay, cells transfected with siCTL or siCARM1 were re-seeded at confluence in 6-well plates, and wounds were performed with a P200 pipette tip. After removing cellular debris by washing cells with PBS, three images of each well were taken. The wounded area was measured by using image J and recorded as A0. The cells were then allowed to migrate back into the wounded area, and three images were taken and the wounded area was measured again 3, 6 and 12 hrs later and recorded as A1, A2 and A3, respectively. Cell migration was presented as wound closure (%) = (wounded area (A0-A1 or A2 or A3)/wounded area A0) × 100%.

For trans-well assay, cells transfected with siCTL or siCARM1 were re-seeded on the top compartment of transwell Boyden chambers (8 μm, Corning, USA) in serum-free media, and then allowed to migrate to the lower compartment contained complete media with 10 % FBS (fetal bovine serum) in a humidified, 5% CO_2_ atmosphere incubator at 37 °C. After 24 hrs, cells that did not migrate into the lower compartment were wiped away with a cotton swab. The inserts were fixed with methanol/acid solution (3:1) for 15 mins and stained with 0.1% crystal violet for 10 mins. After washing with PBS extensively, migrated cells were photographed and quantified using Image J.

For tumor xenograft assay, four groups (4 mice/group) of female BALB/C nude mice (age 4-6 weeks) were subcutaneously implanted with 5×10^6^ of CARM1 (WT) or CARM1 (KO) cells suspended in DMEM medium without FBS. To supplement the estrogen for MCF7 cell proliferation, each nude mouse was brushed with estrogen (E_2_, 10^-2^ M) every 3 days for the duration of the experiments. All mice were euthanized 6 weeks after subcutaneous injection. Tumors were then excised, photographed and weighted. Animals were housed in the Animal Facility at Xiamen University under pathogen-free conditions, following the protocol approved by the Xiamen Animal Care and Use Committee.

### RNA Sequencing (RNA-seq) and Computational Analysis of RNA-seq Data

RNA extraction, DNase I in column digestion and RNA library preparation were performed as described previously 54. Paired-end sequencing was performed with Illumina HiSeq platform at RiboBio Co., Ltd. or Amogene Biotech Co., Ltd.

Three biological repeats were performed and then pooled together. Two pooled sample (six biological repeats) were subjected to library construction and sequencing. Sequencing reads were aligned to hg19 RefSeq database by using Tophat 61 (http://ccb.jhu.edu/software/tophat/index.shtml). Cuffdiff was used to quantify the expression of RefSeq annotated genes with the option -M (reads aligned to repetitive regions were masked) and -u (multiple aligned read are corrected using 'rescue method') 61. Coding genes with FPKM (fragments per kilobase per million mapped reads) larger than or equal to 0.5, either in control or estrogen-treated sample, were included in our analysis. Estrogen-regulated gene program was determined by fold change (FC) of gene FPKM in control and estrogen-treated samples (FC≥1.5). FPKM of a gene was calculated as mapped reads on exons divided by exonic length and the total number of mapped reads. Box plots were generated by R software and significance was determined using Student's t-test. Heat maps were visualized using Java TreeView or R software.

All RNA-seq data were deposited in the Gene Expression Omnibus database under accession GSE124449.

### Chromatin Immunoprecipitation Coupled with High Throughput Sequencing (ChIP-Seq) and Computational Analysis of ChIP-Seq Data

For ChIP assays, cells were maintained in stripping medium (phenol red free) for three days before treating with or without estrogen (E_2_, 10^-7^ M) for 1 hr. Cells were then fixed with 1% formaldehyde (Sigma) for 15 mins at room temperature (RT) (for MED12 (Bethyl Laboratory Inc., A300-774A) and Pol II (Bethyl Laboratory Inc. A300-653A) ChIP), or fixed with disuccinimidyl glutarate (DSG) (2 mM) (Proteochem) for 45 mins at RT, washed twice with PBS and then double-fixed with 1% formaldehyde for another 15 mins at RT (for CARM1 (CST, 12495 and TDRD3 (Proteintech, 13359-1-AP) ChIP). Fixation was stopped by adding glycine (0.125 M) and incubated for 5 mins at RT, followed by washing with PBS twice. Chromatin DNA was sheared to 300~500 bp average in size through sonication. Resultant was immunoprecipitated with anti-MED12, anti-Pol II or anti-CARM1 antibody overnight at 4 ℃, followed by incubation with protein G magnetic beads (Bio-Rad, 161-4023) for an additional 2 hrs. After washing and elution, the protein-DNA complex was reversed by heating at 65 ℃ overnight. Immunoprecipitated DNA was purified by using QIAquick spin columns (Qiagen) and subjected to high throughput sequencing.

For all ChIP-seq done in this manuscript, two biological repeats were performed and then pooled together. ChIP-seq sample preparation and computational analysis of ChIP-seq data were performed as following.

Library construction: the libraries were constructed following Illumina ChIP-seq Sample prep kit. Briefly, ChIP DNA was end-blunted and added with an 'A' base so the adaptors from Illumina with a 'T' can ligate on the ends. Then 200-400 bp fragments are gel-isolated and purified. The library was amplified by 18 cycles of PCR.

Primary analysis of ChIP-Seq datasets: the image analysis and base calling were performed by using Illumina's Genome Analysis pipeline. The sequencing reads were aligned to hg19 Refseq database by using Bowtie2 62 (http://bowtie-bio.sourceforge.net/bowtie2/index.shtml) with default parameters. Both uniquely and multiply aligned reads were kept for downstream analysis (if a read aligned to multiple genomic locations, only one location with the best score was chosen). Clonal amplification was circumvented by allowing maximal one tag for each unique genomic position. The identification of ChIP-seq peaks was performed using HOMER with default parameters 63. Genomic distribution was done by using the default parameters from HOMER with minor modifications, in which promoter peaks were defined as those with peak center falling between 1,000 bp downstream and 5,000 bp upstream of transcript start sites (TSS). To define estrogen-induced CARM1 binding sites, only when fold change (FC) of ChIP-seq tag density of a peak in estrogen treatment versus control was larger than 4, that peak was considered as estrogen specific. Motif analysis was performed using HOMER. Tag density for histograms (25 bp/bin), box plots and heat maps were generated by using HOMER. Box plots were then generated by R software (https://www.r-project.org/) and significance was determined using Student's t test. Heat maps were visualized using Java TreeView 64 (http://jtreeview.sourceforge.net).

ERα ChIP-seq was from GSE45822; H3K27Ac and p300 ChIP-seq were from GSE62229; MED1 ChIP-seq was from GSE60272; MED12 ChIP-seq was from GSE101562; H3K4me3, H3K9me3 and H3K27me3 ChIP-seq were from GSE23701. ChIP-seq was deposited in the Gene Expression Omnibus database under accession GSE124449.

### Mining of the Cancer Genome Atlas (TCGA) Data

Expression data (FPKM) of CARM1, and E2-induced and CARM1-dependent genes in a cohort of TCGA clinical breast samples (tumor: 1,102; normal: 113) was downloaded from GDC Data Portal. Box plots were generated by R software and significance was determined using Student's t-test.

### Generation of CARM1 Knockout Cell Lines Using CRISPR/Cas9 Gene Editing Technology

CARM1 knock out (KO) MCF7 cells were generated by using CRISPR/Cas9 system. Specifically, gRNA sequence (5'-CTCACCATCGGCGACGCGAA-3'), which targets the first exon of CARM1 isoform 1 (NM_199141.2) and 2 (NM_001370088.1) but not alternative exon 1 of isoform 3 (NM_001370089.1), was first cloned into pX330-U6-Chimeric_BB-CBh-hSpCas9 (Addgene, 42230) and confirmed by sequencing, which was named as pX330-gRNA (CARM1). This gRNA was chosen based on our RNA-seq data, which indicated that isoform 2 was the predominant isoform expressed in MCF7 cells, whereas both isoform 1 and 3 barely express as seen from the lack of sequencing reads from the unique exon of isoform 1 or 3. MCF7 cells were then transfected with pX330-gRNA (CARM1) and pIREShyg3 vector (Clontech, 631620) (for selection purpose), followed by hygromycin (0.2 mg/mL) selection. Single colonies were subjected to immunoblotting to select knock-out ones.

### Global Mapping of CARM1 Substrates

#### Cell labeling by SILAC (Stable Isotope Labeling by Amino Acids in Cell Culture)

Wild type and CARM1 KO MCF7 cells were grown in SILAC DMEM (Invitrogen) supplemented with L-lysine/arginine (Sigma) and L-lysine/arginine-U-13C6 (Cambridge Isotope Laboratories), respectively, together with 10% dialyzed FBS, L-glutamine and penicillin/streptomycin for 2 weeks. Cells were cultured at 37°C in a humidified atmosphere containing 5% CO2. Cells were harvested, centrifuged (5 mins, 500 g), rinsed twice with ice-cold phosphate-buffered saline (PBS) and stored at -80°C briefly before cell lysis.

#### Cell lysis and sample preparation

Cells were lysed in ten volumes of modified SDT buffer (0.1 M Tris HCL, pH 8.5, 0.1 M DTT, 1% SDS, 1% SDC) and incubated at 95°C for 5 mins. The lysate was sonicated to shear genomic DNA, and clarified by centrifugation at 20,000 g for 15 min at 20°C. The supernatant was transferred to ultrafiltration units (Millipore, Amicon Ultra 15 Ultracel 10 KD) and centrifuged at 4,000 g for 40 mins. After centrifugation, the concentrates were mixed with 2 mL of 50 mM iodoacetamide in UA solution (8 M urea, 100 mM Tris-HCl pH 8.5) and incubated in darkness at room temperature (RT) for 30 mins followed by centrifugation for 30 mins. After alkylation, the filter units were washed four times with 10 ml UA buffer and two times 10 mL of 50 mM ammonium bicarbonate by centrifugation at 4,000 g. Proteins were then digested with Lys-C (1:100, w/w, Wako) for 6 hrs at 37°C and trypsin (1:50, w/w, Promega) overnight at 37°C. The resulting peptide mixture was acidified (pH 2) with formic acid, loaded onto Sep-Pak tC18 cartridges (Waters), desalted and eluted with 70% acetonitrile. The eluted peptides were lyophilized and stored at -80°C before analysis.

#### Offline High-pH fractionation

100 μg peptides (for proteome analysis) or 15 mg peptides (for methylome analysis) were off-line fractionated by bRP (basic Reverse Phase) using a Waters XBridge BEH C18 5 μm 4.6 × 250 mm column (Waters) or XBridge BEH C18 10 μm 10 × 250 mm column (Waters) on an Ultimate 3000 high-pressure liquid chromatography (HPLC) system (Dionex, Sunnyvale, CA, USA) operating at 1 mL/min or 2.5 mL/min. Buffer A (5 mM ammonium formate, pH 10) and buffer B (5 mM ammonium formate, pH 10, 90% (v/v) ACN) were adjusted to pH 10 with ammonium hydroxide. Peptides were separated by a linear gradient from 5% B to 35% B in 54 mins followed by a linear increase to 70% B in 6 mins. A total of 60 fractions were collected. For comprehensive proteomic analysis, the 60 fractions were concatenated to 20. For comprehensive methylome analysis, the 60 fractions were concatenated to 10. All the concentrated fractions were lyophilized.

#### Enrichment of mono- and asymmetrical di-methylated arginine peptides

The lyophilized peptides were dissolved by 600 μl 1×PTMScan IAP Buffer (CST). Two vials of PTMScan mono-methyl arginine motif [mme-RG] immunoaffinity beads (CST) were divided and equal amounts were incubated with the 10 dissolved fractions for 4 hrs at 4°C. After enrichment with mono-methylated antibodies, the supernatant from the 10 fractions were concatenated to 5. One vial of PTMScan asymmetric di-methyl arginine motif [adme-R] immunoaffinity beads (CST) were divided and equal amounts were incubated with the 5 fractions for 4 hrs at 4°C. All the immunoprecipitates were washed three times in ice-cold immunoprecipitation buffer followed by three washes in water, and modified peptides were eluted with 2 × 50 μl of 0.15% trifluoroacetic acid in Milli-Q water. Peptide eluates were desalted on reversed-phase C18 StageTips and dried by speed vacuum at 45°C.

#### LC-MS/MS analysis

All MS experiments were performed on a nanoscale EASY-nLC 1200UHPLC system (Thermo Fisher Scientific) connected to an Orbitrap Fusion Lumos equipped with a nanoelectrospray source (Thermo Fisher Scientific). Mobile phase A contained 0.1% formic acid (v/v) in water; mobile phase B contained 0.1% formic acid in 80% acetonitrile (ACN). The peptides were dissolved in 0.1% formic acid (FA) with 2% acetonitrile and separated on a RP-HPLC analytical column (75 μm×25 cm) packed with 2 μm C18 beads (Thermo Fisher Scientific) using a linear gradient ranging from 5% to 22% ACN in 90 mins and followed by a linear increase to 35% B in 20 mins at a flow rate of 300 nL/min. The Orbitrap Fusion Lumos acquired data in a data-dependent manner alternating between full-scan MS and MS2 scans. The spray voltage was set at 2.2 kV and the temperature of ion transfer capillary was 300°C. The MS spectra (350-1500 m/z) were collected with 120,000 resolutions, AGC of 4 × 10^5^, and 50 ms maximal injection time. Selected ions were sequentially fragmented in a 3 seconds (s) cycle by HCD with 30% normalized collision energy, specified isolated windows 1.6m/z, 30,000 resolutions. AGC of 5 × 10^4^ and 150 ms maximal injection time were used. Dynamic exclusion was set to 15 s. Unassigned ions or those with a charge of 1+ and >7+ were rejected for MS/MS.

#### Mass spectrometry data analysis

Raw data was processed using Proteome Discoverer (PD, version 2.1), and MS/MS spectra were searched against the reviewed SwissProt human proteome database. All searches were carried out with precursor mass tolerance of 20 ppm, fragment mass tolerance of 0.02 Da, oxidation (Met) (+15.9949 Da), methylation (Arg, Lys) (+14.0266 Da), dimethylation (Arg, Lys) (+28.0532 Da), trimethylation (Lys) (+42.0470 Da) and acetylation (protein N-terminus) (+42.0106 Da) as variable modifications, carbamidomethylation (Cys) (+57.0215 Da) as fixed modification and three trypsin missed cleavages allowed. Only peptides with at least six amino acids in length were considered. The peptide and protein identifications were filtered by PD to control the false discovery rate (FDR) <1%. At least one unique peptide was required for protein identification.

#### Bioinformatics analysis

Mono- and di-methylation sites were first extracted. Proteins with arginine methylation sites on which methylation signals were abolished in CARM1 KO cells were considered as “CARM1 methylome”. Motif analysis was performed by using the IceLogo web server taking 5 amino acids (AAs) upstream and downstream of the arginine methylation site identified (11 AAs in total). Mutation frequency at or in the vicinity of CARM1-dependent arginine sites (5 AAs upstream and downstream) was calculated based on the COSMIC database. Similar analysis was performed for any arginine site in the human proteome, and P value was calculated by the Fisher's exact test. Gene Ontology (GO) analysis on CARM1 substrates was performed using the Metascape web server.

### SILAC, Affinity Purification, In Gel Digestion and LC-MS/MS Analysis of MED12 methylation

Wild type and CARM1 KO MCF7 cells were labeled as described above and then infected with Lenti-viral vectors expressing pBobi-Flag-MED12 (1616-2177)-HA for 48 hrs before adding estrogen (10^-7^ M) for 1 hr. Cells were then lysed in a buffer containing 50 mM Tris-HCl (pH 7.4), 420 mM NaCl, 1 mM EDTA and 1% Triton X-100, pooled and subjected to affinity purification by using anti-Flag M2-agarose, washed extensively with a buffer containing 50 mM Tris-HCl (pH 7.4), 420 mM NaCl, 1 mM EDTA and 1% Triton X-100, and eluted with 3X Flag peptides (Sigma). Elutes were then separated by SDS-PAGE gel and stained with Coommassie blue. The band corresponding to MED12 was cut, de-stained and subjected to in gel digestion and LC-MS/MS analysis following the protocol described below.

The gel slices were cut to cubes (1X1 mm) and transferred to Lobind tubes (Eppendorf), and 300 μL LC-MS water was then added for 15 mins at room temperature (RT) with agitation. The same volume of acetonitrile (ACN) was added and incubated for 15 mins. The supernatant was discarded and 100 μL LC-MS ACN was then added for 5 mins at RT. Samples were dried in a Speedvac (Eppendorf) and then reduced by mixing with 200 μL of 100 mM ammonium bicarbonate/10 mM DTT and incubated at 56 °C for 30 mins. The liquid was removed and 200 μL of 100 mM ammonium bicarbonate/50 mM iodoacetamide (IAA) was added to gel pieces and incubated at RT in the dark for 30 mins. After removal of the supernatant and one wash with 300 μL 100 mM ammonium bicarbonate for 15 mins, same volume of ACN was added to dehydrate the gel pieces. The solution was then removed and samples were dried in a Speedvac. For digestion, enough solution of ice-cold trypsin (0.01 µg/μL) in 20 mM ammonium bicarbonate was added to cover the gel pieces and set on ice for 30 mins. After complete rehydration, the excess trypsin solution was removed, replaced with 20 mM ammonium bicarbonate to completely cover the gel pieces, and left overnight at 37°C. The peptides were extracted twice with 50 μL of 50% ACN/1% formic acid (FA) and vortex at RT for 30 mins. All extracts were pooled and dried in a Speedvac, followed by using ZipTips to purify and concentrate peptides for LC-MS/MS analysis. MS experiments were performed on a nanoscale UHPLC system (EASY-nLC1000, Proxeon Biosystems) connected to an Orbitrap Q-Exactive equipped with a nanoelectrospray source (Thermo Fisher Scientific) as described previously 54.

## Supplementary Material

Supplementary figures and table legends.

Supplementary table 1.

Supplementary table 2.

Supplementary table 3.

## References

[1] Perou CM, Jeffrey SS, van de Rijn M, Rees CA, Eisen MB, Ross DT (1999). Distinctive gene expression patterns in human mammary epithelial cells and breast cancers. Proceedings of the National Academy of Sciences of the United States of America.

[2] Perou CM, Sorlie T, Eisen MB, van de Rijn M, Jeffrey SS, Rees CA (2000). Molecular portraits of human breast tumours. Nature.

[3] Foulds CE, Feng Q, Ding C, Bailey S, Hunsaker TL, Malovannaya A (2013). Proteomic analysis of coregulators bound to ERalpha on DNA and nucleosomes reveals coregulator dynamics. Molecular cell.

[4] Dou XW, Liang YK, Lin HY, Wei XL, Zhang YQ, Bai JW (2017). Notch3 Maintains Luminal Phenotype and Suppresses Tumorigenesis and Metastasis of Breast Cancer via Trans-Activating Estrogen Receptor-alpha. Theranostics.

[5] Hervouet E, Cartron PF, Jouvenot M, Delage-Mourroux R (2013). Epigenetic regulation of estrogen signaling in breast cancer. Epigenetics.

[6] Zheng Y, Zeng Y, Qiu R, Liu R, Huang W, Hou Y (2018). The Homeotic Protein SIX3 Suppresses Carcinogenesis and Metastasis through Recruiting the LSD1/NuRD(MTA3) Complex. Theranostics.

[7] Gary JD, Clarke S (1998). RNA and protein interactions modulated by protein arginine methylation. Progress in nucleic acid research and molecular biology.

[8] Larsen SC, Sylvestersen KB, Mund A, Lyon D, Mullari M, Madsen MV (2016). Proteome-wide analysis of arginine monomethylation reveals widespread occurrence in human cells. Science signaling.

[9] Shishkova E, Zeng H, Liu F, Kwiecien NW, Hebert AS, Coon JJ (2017). Global mapping of CARM1 substrates defines enzyme specificity and substrate recognition. Nature communications.

[10] Guo A, Gu H, Zhou J, Mulhern D, Wang Y, Lee KA (2014). Immunoaffinity enrichment and mass spectrometry analysis of protein methylation. Molecular & cellular proteomics: MCP.

[11] Sylvestersen KB, Horn H, Jungmichel S, Jensen LJ, Nielsen ML (2014). Proteomic analysis of arginine methylation sites in human cells reveals dynamic regulation during transcriptional arrest. Molecular & cellular proteomics: MCP.

[12] Geoghegan V, Guo A, Trudgian D, Thomas B, Acuto O (2015). Comprehensive identification of arginine methylation in primary T cells reveals regulatory roles in cell signalling. Nature communications.

[13] Bedford MT, Clarke SG (2009). Protein arginine methylation in mammals: who, what, and why. Molecular cell.

[14] Wolf SS (2009). The protein arginine methyltransferase family: an update about function, new perspectives and the physiological role in humans. Cellular and molecular life sciences: CMLS.

[15] Blanc RS, Richard S (2017). Arginine Methylation: The Coming of Age. Molecular cell.

[16] Yang Y, Bedford MT (2013). Protein arginine methyltransferases and cancer. Nature reviews Cancer.

[17] Poulard C, Corbo L, Le Romancer M (2016). Protein arginine methylation/demethylation and cancer. Oncotarget.

[18] Chen D, Ma H, Hong H, Koh SS, Huang SM, Schurter BT (1999). Regulation of transcription by a protein methyltransferase. Science.

[19] Torres-Padilla ME, Parfitt DE, Kouzarides T, Zernicka-Goetz M (2007). Histone arginine methylation regulates pluripotency in the early mouse embryo. Nature.

[20] Wu Q, Bruce AW, Jedrusik A, Ellis PD, Andrews RM, Langford CF (2009). CARM1 is required in embryonic stem cells to maintain pluripotency and resist differentiation. Stem cells.

[21] Yadav N, Lee J, Kim J, Shen J, Hu MC, Aldaz CM (2003). Specific protein methylation defects and gene expression perturbations in coactivator-associated arginine methyltransferase 1-deficient mice. Proceedings of the National Academy of Sciences of the United States of America.

[22] Kim YR, Lee BK, Park RY, Nguyen NT, Bae JA, Kwon DD (2010). Differential CARM1 expression in prostate and colorectal cancers. BMC cancer.

[23] Elakoum R, Gauchotte G, Oussalah A, Wissler MP, Clement-Duchene C, Vignaud JM (2014). CARM1 and PRMT1 are dysregulated in lung cancer without hierarchical features. Biochimie.

[24] Hong H, Kao C, Jeng MH, Eble JN, Koch MO, Gardner TA (2004). Aberrant expression of CARM1, a transcriptional coactivator of androgen receptor, in the development of prostate carcinoma and androgen-independent status. Cancer.

[25] Frietze S, Lupien M, Silver PA, Brown M (2008). CARM1 regulates estrogen-stimulated breast cancer growth through up-regulation of E2F1. Cancer research.

[26] Majumder S, Liu Y, Ford OH 3rd, Mohler JL, Whang YE (2006). Involvement of arginine methyltransferase CARM1 in androgen receptor function and prostate cancer cell viability. The Prostate.

[27] Al-Dhaheri M, Wu J, Skliris GP, Li J, Higashimato K, Wang Y (2011). CARM1 is an important determinant of ERalpha-dependent breast cancer cell differentiation and proliferation in breast cancer cells. Cancer research.

[28] Osada S, Suzuki S, Yoshimi C, Matsumoto M, Shirai T, Takahashi S (2013). Elevated expression of coactivator-associated arginine methyltransferase 1 is associated with early hepatocarcinogenesis. Oncology reports.

[29] Cheng H, Qin Y, Fan H, Su P, Zhang X, Zhang H (2013). Overexpression of CARM1 in breast cancer is correlated with poorly characterized clinicopathologic parameters and molecular subtypes. Diagnostic pathology.

[30] Habashy HO, Rakha EA, Ellis IO, Powe DG (2013). The oestrogen receptor coactivator CARM1 has an oncogenic effect and is associated with poor prognosis in breast cancer. Breast cancer research and treatment.

[31] Covic M, Hassa PO, Saccani S, Buerki C, Meier NI, Lombardi C (2005). Arginine methyltransferase CARM1 is a promoter-specific regulator of NF-kappaB-dependent gene expression. The EMBO journal.

[32] Jansson M, Durant ST, Cho EC, Sheahan S, Edelmann M, Kessler B (2008). Arginine methylation regulates the p53 response. Nature cell biology.

[33] Wang L, Zhao Z, Meyer MB, Saha S, Yu M, Guo A (2014). CARM1 methylates chromatin remodeling factor BAF155 to enhance tumor progression and metastasis. Cancer cell.

[34] Naeem H, Cheng D, Zhao Q, Underhill C, Tini M, Bedford MT (2007). The activity and stability of the transcriptional coactivator p/CIP/SRC-3 are regulated by CARM1-dependent methylation. Molecular and cellular biology.

[35] Liu F, Ma F, Wang Y, Hao L, Zeng H, Jia C (2017). PKM2 methylation by CARM1 activates aerobic glycolysis to promote tumorigenesis. Nature cell biology.

[36] Yi P, Wang Z, Feng Q, Chou CK, Pintilie GD, Shen H (2017). Structural and Functional Impacts of ER Coactivator Sequential Recruitment. Molecular cell.

[37] Davis MB, Liu X, Wang S, Reeves J, Khramtsov A, Huo D (2013). Expression and sub-cellular localization of an epigenetic regulator, co-activator arginine methyltransferase 1 (CARM1), is associated with specific breast cancer subtypes and ethnicity. Molecular cancer.

[38] Xu W, Cho H, Kadam S, Banayo EM, Anderson S, Yates JR 3rd (2004). A methylation-mediator complex in hormone signaling. Genes & development.

[39] Xu W, Chen H, Du K, Asahara H, Tini M, Emerson BM (2001). A transcriptional switch mediated by cofactor methylation. Science.

[40] Cheng D, Vemulapalli V, Lu Y, Shen J, Aoyagi S, Fry CJ (2018). CARM1 methylates MED12 to regulate its RNA-binding ability. Life science alliance.

[41] Yang Y, Lu Y, Espejo A, Wu J, Xu W, Liang S (2010). TDRD3 is an effector molecule for arginine-methylated histone marks. Molecular cell.

[42] Ceschin DG, Walia M, Wenk SS, Duboe C, Gaudon C, Xiao Y (2011). Methylation specifies distinct estrogen-induced binding site repertoires of CBP to chromatin. Genes & development.

[43] Liu Y, Li J, Shang Y, Guo Y, Li Z (2019). CARM1 contributes to skeletal muscle wasting by mediating FoxO3 activity and promoting myofiber autophagy. Experimental cell research.

[44] Maurer-Stroh S, Dickens NJ, Hughes-Davies L, Kouzarides T, Eisenhaber F, Ponting CP (2003). The Tudor domain 'Royal Family': Tudor, plant Agenet, Chromo, PWWP and MBT domains. Trends in biochemical sciences.

[45] Friesen WJ, Massenet S, Paushkin S, Wyce A, Dreyfuss G (2001). SMN, the product of the spinal muscular atrophy gene, binds preferentially to dimethylarginine-containing protein targets. Molecular cell.

[46] Brahms H, Meheus L, de Brabandere V, Fischer U, Luhrmann R (2001). Symmetrical dimethylation of arginine residues in spliceosomal Sm protein B/B' and the Sm-like protein LSm4, and their interaction with the SMN protein. Rna.

[47] Yun M, Wu J, Workman JL, Li B (2011). Readers of histone modifications. Cell research.

[48] Sims RJ 3rd, Rojas LA, Beck DB, Bonasio R, Schuller R, Drury WJ 3rd (2011). The C-terminal domain of RNA polymerase II is modified by site-specific methylation. Science.

[49] Li W, Notani D, Ma Q, Tanasa B, Nunez E, Chen AY (2013). Functional roles of enhancer RNAs for oestrogen-dependent transcriptional activation. Nature.

[50] Uhlmann T, Geoghegan VL, Thomas B, Ridlova G, Trudgian DC, Acuto O (2012). A method for large-scale identification of protein arginine methylation. Molecular & cellular proteomics: MCP.

[51] Hu H, Qian K, Ho MC, Zheng YG (2016). Small Molecule Inhibitors of Protein Arginine Methyltransferases. Expert opinion on investigational drugs.

[52] Wong CC, Kang W, Xu J, Qian Y, Luk STY, Chen H (2019). Prostaglandin E(2) induces DNA hypermethylation in gastric cancer in vitro and in vivo. Theranostics.

[53] Zhao T, Bao Y, Gan X, Wang J, Chen Q, Dai Z (2019). DNA methylation-regulated QPCT promotes sunitinib resistance by increasing HRAS stability in renal cell carcinoma. Theranostics.

[54] Gao WW, Xiao RQ, Zhang WJ, Hu YR, Peng BL, Li WJ (2018). JMJD6 Licenses ERalpha-Dependent Enhancer and Coding Gene Activation by Modulating the Recruitment of the CARM1/MED12 Co-activator Complex. Mol Cell.

[55] Wang J, Wang L, Feng G, Wang Y, Li Y, Li X (2018). Asymmetric Expression of LincGET Biases Cell Fate in Two-Cell Mouse Embryos. Cell.

[56] Bao J, Rousseaux S, Shen J, Lin K, Lu Y, Bedford MT (2018). The arginine methyltransferase CARM1 represses p300*ACT*CREMtau activity and is required for spermiogenesis. Nucleic acids research.

[57] Hupalowska A, Jedrusik A, Zhu M, Bedford MT, Glover DM, Zernicka-Goetz M (2018). CARM1 and Paraspeckles Regulate Pre-implantation Mouse Embryo Development. Cell.

[58] Wang YP, Zhou W, Wang J, Huang X, Zuo Y, Wang TS (2016). Arginine Methylation of MDH1 by CARM1 Inhibits Glutamine Metabolism and Suppresses Pancreatic Cancer. Molecular cell.

[59] Shin HJ, Kim H, Oh S, Lee JG, Kee M, Ko HJ (2016). AMPK-SKP2-CARM1 signalling cascade in transcriptional regulation of autophagy. Nature.

[60] Cheng D, Cote J, Shaaban S, Bedford MT (2007). The arginine methyltransferase CARM1 regulates the coupling of transcription and mRNA processing. Molecular cell.

[61] Trapnell C, Roberts A, Goff L, Pertea G, Kim D, Kelley DR (2012). Differential gene and transcript expression analysis of RNA-seq experiments with TopHat and Cufflinks. Nature protocols.

[62] Langmead B, Salzberg SL (2012). Fast gapped-read alignment with Bowtie 2. Nat Methods.

[63] Heinz S, Benner C, Spann N, Bertolino E, Lin YC, Laslo P (2010). Simple combinations of lineage-determining transcription factors prime cis-regulatory elements required for macrophage and B cell identities. Molecular cell.

[64] Saldanha AJ (2004). Java Treeview-extensible visualization of microarray data. Bioinformatics.

