# Supplementary material for: A hypermethylation strategy utilized by enhancer-bound CARM1 to promote estrogen receptor α-dependent transcriptional activation and breast carcinogenesis
Source: Theranostics. 2020 Feb 10;10(8):3451–73. doi: 10.7150/thno.39241 (PMC7069091; doi:10.7150/thno.39241)
Supplement: Supplementary file 1 — Supplementary figures and table legends. [file thnov10p3451s1.pdf]

Supplementary Information

Supplementary Figure Legends

Figure S1. CARM1 is required for estrogen-induced gene transcriptional activation.

(A) Box plot showing the expression of CARM1 (FPKM) in a cohort of clinical breast cancer (n=1,102) and normal (n=113) samples from TCGA (The Cancer Genome Atlas).

(B, C) Kaplan Meier survival analyses for DMFS (distal metastasis free survival) (n=1,747) and OS (overall survival) (n=1,402) of breast cancer patients using CARM1 as input.

(D, E) RNA-seq as described in Figure 1A for specific genes were shown as indicated.

(F) Correlation of CARM1's effects on whole transcriptome in response to estrogen between two RNA-seq biological repeats (n=11,816).

(G) Correlation of CARM1 effects on whole transcriptome in response to estrogen based on RNA-seq between two independent siRNAs targeting CARM1 (siCARM1 (1) and siCARM1 (2)) (n=12,234).

(H, I) MCF7 cells as described in (G) were subjected to immunoblotting (IB) (H) and RT-qPCR (I) analysis to examine the levels of indicated protein and mRNA, respectively. Actin was served as a loading control.

(J) MCF7 cells as described in Figure 1E were subjected to RT-qPCR analysis to examine the expression of genes as indicated.

(K) MCF7 cells as described in Figure 1E were subjected to immunoblotting (IB) analysis to examine the protein levels of CARM1. Actin was served as a loading control.

(L) Genomic DNA was extracted from CARM1 knockout cells, followed by PCR using specific primer sets spanning gRNA targeting region (boxed in blue). The resultant PCR products were subjected to Sanger sequencing as shown. Nucleotide being inserted was highlighted in red.

(M) MCF7 cells as described in Figure 1F were subjected to immunoblotting (IB) analysis to examine the protein levels of CARM1 by using anti-specific CARM1 antibodies from two independent vendors as indicated. Actin was served as a loading control.

(N, O) Pol II ChIP-seq signals across specific genes as indicated. Units are mean tags per bin for 16 bins across the transcribed gene region with 2 kb upstream (4 bins of 500 bp each) and 5 kb downstream flanking regions (10 bins of 500 bp each).

(P, Q) Pol II ChIP-seq tag density distribution centered on cognate enhancers of specific genes as indicated (± 4,000 bp).

(R, S) Heat map (R) and box plots (S) representation of the expression levels (FPKM) for genes induced by estrogen and dependent on CARM1 in the cohort of clinical samples, both normal and tumor, as described in (A).

Figure S2. CARM1 is recruited onto ERα-bound active enhancers upon estrogen stimulation.

(A) MCF7 cells treated with or without estrogen (E_2, 10^{-7} M, 1 hr) were subjected to ChIP with anti-CARM1 antibody followed by qPCR analysis with primers specifically targeting enhancer (e) regions as indicated. ChIP signals were presented as percentage of inputs (± s.e.m.).

(B) ChIP-seq tag distribution, including ERα, H3K4me1, H3K4me2, H3K4me3, H3K27Ac, P300, MED1, MED12, H3K9me3 and H3K27me3, centered on all of its own sites (left panels) or estrogen-induced CARM1 sites (right panels) (± 4,000 bp).

(C) Genome browser views of CARM1, ERα, H3K4me1, H3K4me2, H3K4me3, H3K27Ac, P300, MED1, MED12, H3K9me3 and H3K27me3 ChIP-seq in the presence and absence of estrogen on selected active enhancer regions, such as the ones in the vicinity of estrogen-induced coding gene *NRIP1* (left panel on the top), *PGR* (right panel on the top), *SIAH2* (left panel on the bottom) and

APOA1 (right panel on the bottom), were shown. Boxed regions indicated active enhancers. ChIP-seq views, except for CARM1, on *SIAH2* have been shown in our previous study [1].

Figure S3. Global mapping of CARM1 substrates.

(A) Experimental workflow for global mapping of CARM1 substrates. FASP: filter aided sample preparation.

(B) The distribution of proteins with different number of arginine methylation sites. All the proteins with more than ten methylation sites were shown in oval.

(C) Schematic representation of domain architecture of representative proteins with clustered arginine methylation sites in the "CARM1 methylome". Arginine methylation sites were shown by matchsticks. CID: CTD-interacting domain; CTD: Spt5 C-terminal domain; G-patch: glycine rich nucleic binding domain; DRBM: double strand RNA-binding domain; PHD: the plant homeodomain zinc finger; TFS2M: transcription factor S-II (TFIIS) domain in the central regions of transcription elongation factor S-II (and elsewhere); RING: ring finger; BBC: B-Box C-terminal domain; BBOX: B-Box-type zinc finger; BROMO: bromo domain; TFS2N: transcription factor S-II (TFIIS) domain in the N-terminus of transcription elongation factor S-II (and elsewhere); ZnF_C3H1: (CCCH) type Zinc finger (Znf) domain; KH: K homology RNA-binding domain; IF rod: Intermediate filament (IF) rod domain; WW: domain with 2 conserved Trp (W) residues; FF: a novel motif that often accompanies WW domains and contains two conserved Phe (F) residues.

(D, E) Rates of somatic mutations at (D) and in the vicinity of (E) CARM1 methylation sites in human cancers. Rates of somatic mutations at and in the vicinity of (±5 nucleotides) CARM1-dependent methylation sites (n=589) were significantly higher than those of arginines in the proteome (P = 1.62e-11 and 7.42e-45, respectively).

Figure S4. CARM1 hypermethylates MED12.

(A) Human MED12 C-terminus (amino acid (aa) 1616-2177). Arginine residues with mono-methylation only, di-methylation only and both mono- and di-methylation were highlighted in red, green and yellow, respectively.

Figure S5. CARM-mediated methylation is involved in estrogen-induced gene transcriptional activation.

(A) MCF7 cells as described in Figure 5F were subjected to RT-qPCR analysis to examine the expression of CARM1.

(B) MCF7 cells as described in Figure 5F were subjected to immunoblotting analysis to examine the expression of MED12 and CARM1. Actin was served as a loading control.

(C) Correlation of MED12's effects on transcriptome in response to estrogen based on RNA-seq between two independent siRNAs targeting MED12 (siMED12 (1) and siMED12 (2)).

(D) Correlation of CARM1's effects on MED12 binding based on ChIP-seq between two independent siRNAs targeting CARM1 (siCARM1 (1) and siCARM1 (2)).

(E, F) MCF7 cells were transfected with control siRNA (siCTL) or two independent siRNAs specific against CARM1 (siCARM1 (1) and siCARM1 (2)) in stripping medium for three days, and treated with or without estrogen (E_2, 10^{-7} M, 6 hrs), followed by RT-qPCR (E) and immunoblotting (IB) (F) analysis to examine the mRNA and protein levels of MED12, respectively. Actin was served as a loading control.

(G) MCF7 cells as described in Figure 5J were subjected to RT-qPCR analysis to examine the fold induction by estrogen for selected genes as indicated (± s.e.m., **$P<0.01$, ***$P<0.001$).

(H, I) MCF7 cells as described in Figure 5J were subjected to RT-qPCR analysis to examine the expression of CARM1 (H) and MED12 (I) (± s.e.m., ***$P<0.001$).

Figure S6. TDRD3 is required for estrogen-induced gene transcriptional activation.

(A-C) MCF7 cells as described in Figure 6G were subjected to qPCR analysis to examine the expression of selected estrogen-induced genes as indicated.

(D-F) MCF7 cells as described in Figure 6I were subjected to qPCR analysis to examine the expression of selected estrogen-induced genes as indicated.

Figure S7. CARM1 is required for estrogen-induced breast cancer cell growth.

(A) WT and CARM1 KO MCF7 cells were subjected to cell proliferation assay (± s.e.m., ***P<0.001).

(B) MCF7 cells as described in (A) were subjected to FACS analysis.

(C, D) T47D (C) and BT474 (D) cells transfected with siCTL or siCARM1 were subjected to cell proliferation assay (± s.e.m., *P<0.01, **P<0.01, ***P<0.001).

(E, F) T47D (E) and BT474 (F) cells transfected with siCTL or siCARM1 were subjected to colony formation assay.

Supplementary Table Legend

Table S1. Global mapping of CARM1 substrates. The total number of arginine methylation sites (the sum of mono- and asymmetrical di-methylation sites after removing duplicates), and the number of mono- or asymmetrical di-methyl arginine methylation sites for proteins which had arginine methylation sites identified (sheet 1), had arginine methylation sites on which methylation signals decreased at least two-fold (sheet 2) or completely abolished (sheet 3), and had all its methylation sites abolished (sheet 4) were shown.

Table S2. MS2 spectrum of arginine-methylated peptides in MED12. MS2 spectrum of methylated arginine residues in MED12 C-terminus as described in Figure 4D were shown as indicated.

Table S3. Sequence information for all qPCR primers used in the current study. Sequence information of qPCR primers designed to detect the expression of *GREB1*, *NRIP1*, *PGR*, *SIAH2*, *TSKU*, *APOA1*, *ERα*, *PRSS23*, *PDCD2L*, *PSAT1*, *CAD*, *CARM1*, *MED12*, *TDRD3*, *SMN*, *SND1* and *SPF30* mRNA, and *GREB1*, *NRIP1*, *PGR*, *SIAH2*, *TSKU* and *APOA1*enhancer RNA (eRNA), and to detect the binding of CARM1 on *TFF1* enhancer (e), *GREB1* (e), *FOXC1* (e), *PGR* (e), *SIAH2* (e), *SMAD7* (e), *TSKU* (e) and *APOA1* (e) regions were shown. F: forward; R: reverse.

Supplementary Reference

1. Gao WW, Xiao RQ, Zhang WJ, Hu YR, Peng BL, Li WJ, et al. JMJD6 Licenses ERalpha-Dependent Enhancer and Coding Gene Activation by Modulating the Recruitment of the CARM1/MED12 Co-activator Complex. Mol Cell. 2018; 70: 340-57 e8.

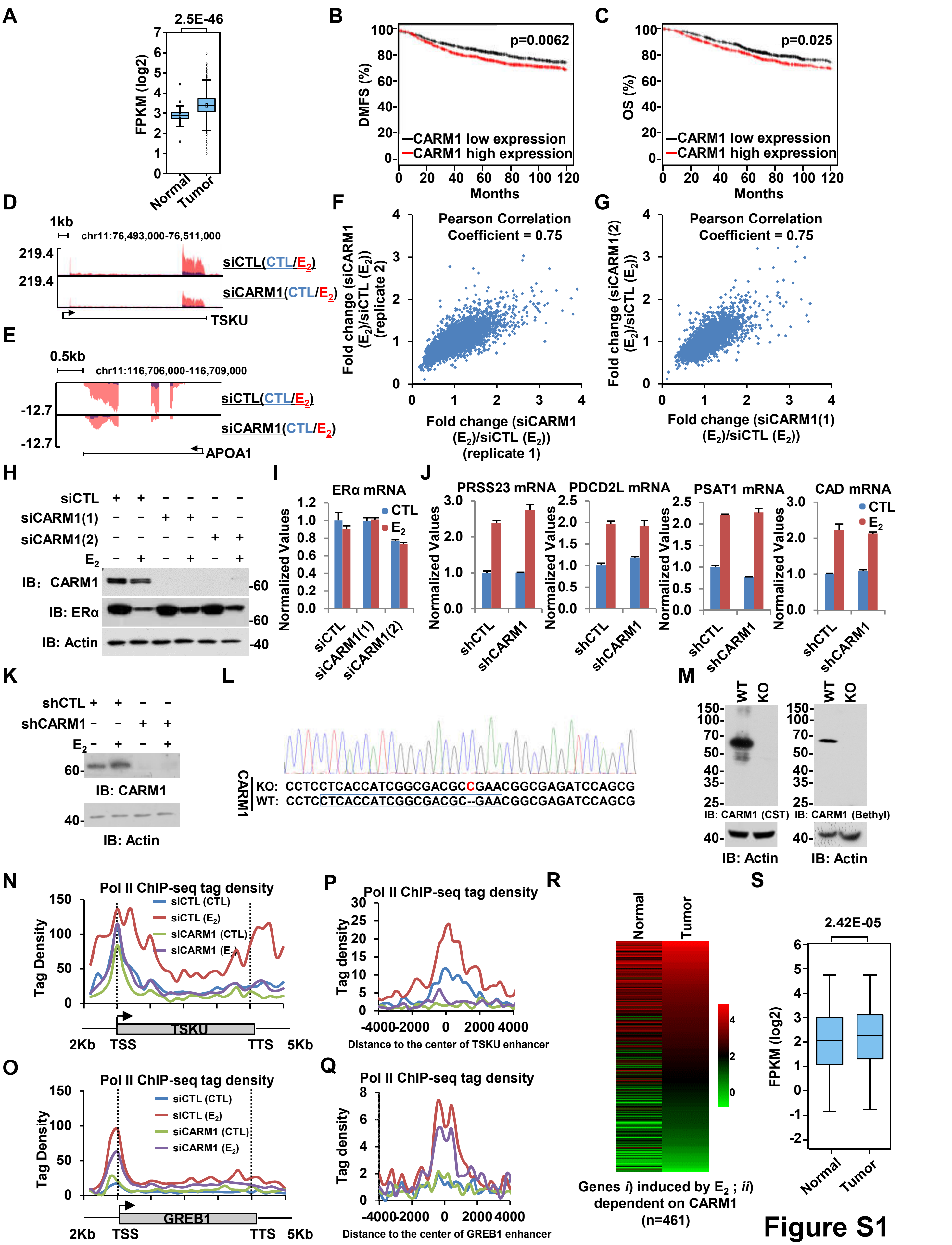
A
2.5E-46
FPKM (log2)
Normal
Tumor
B
DMFS (%)
p=0.0062
CARM1 low expression
CARM1 high expression
Months
C
OS (%)
p=0.025
CARM1 low expression
CARM1 high expression
Months
D
1kb
chr11:76,493,000-76,511,000
219.4
siCTL(CTL/E2)
siCARM1(CTL/E2)
TSKU
E
0.5kb
chr11:116,706,000-116,709,000
-12.7
siCTL(CTL/E2)
siCARM1(CTL/E2)
APOA1
F
Pearson Correlation Coefficient = 0.75
Fold change (siCARM1 (E2)/siCTL (E2)) (replicate 2)
Fold change (siCARM1 (E2)/siCTL (E2)) (replicate 1)
G
Pearson Correlation Coefficient = 0.75
Fold change (siCARM1(2) (E2)/siCTL (E2))
Fold change (siCARM1(1) (E2)/siCTL (E2))
H
siCTL
siCARM1(1)
siCARM1(2)
E2
IB: CARM1
IB: ERα
IB: Actin
-60
-60
-40
I
ERα mRNA
CTL
E2
Normalized Values
siCTL
siCARM1(1)
siCARM1(2)
J
PRSS23 mRNA
PDCD2L mRNA
PSAT1 mRNA
CAD mRNA
Normalized Values
shCTL
shCARM1
K
shCTL
shCARM1
E2
60-
IB: CARM1
40-
IB: Actin
L
CARM1
KO: CCTCCTCACCATCGGCGACGCCGAACGGCGAGATCCAGCG
WT: CCTCCTCACCATCGGCGACGC--GAACGGCGAGATCCAGCG
M
WT
KO
IB: CARM1 (CST)
IB: CARM1 (Bethyl)
IB: Actin
IB: Actin
N
Pol II ChIP-seq tag density
siCTL (CTL)
siCTL (E2)
siCARM1 (CTL)
siCARM1 (E2)
Tag Density
TSKU
2Kb
TSS
TTS
5Kb
O
Pol II ChIP-seq tag density
siCTL (CTL)
siCTL (E2)
siCARM1 (CTL)
siCARM1 (E2)
Tag Density
GREB1
2Kb
TSS
TTS
5Kb
P
Pol II ChIP-seq tag density
Tag density
Distance to the center of TSKU enhancer
Q
Pol II ChIP-seq tag density
Tag density
Distance to the center of GREB1 enhancer
R
Normal
Tumor
Genes i) induced by E2 ; ii) dependent on CARM1 (n=461)
S
2.42E-05
FPKM (log2)
Normal
Tumor

Figure S1

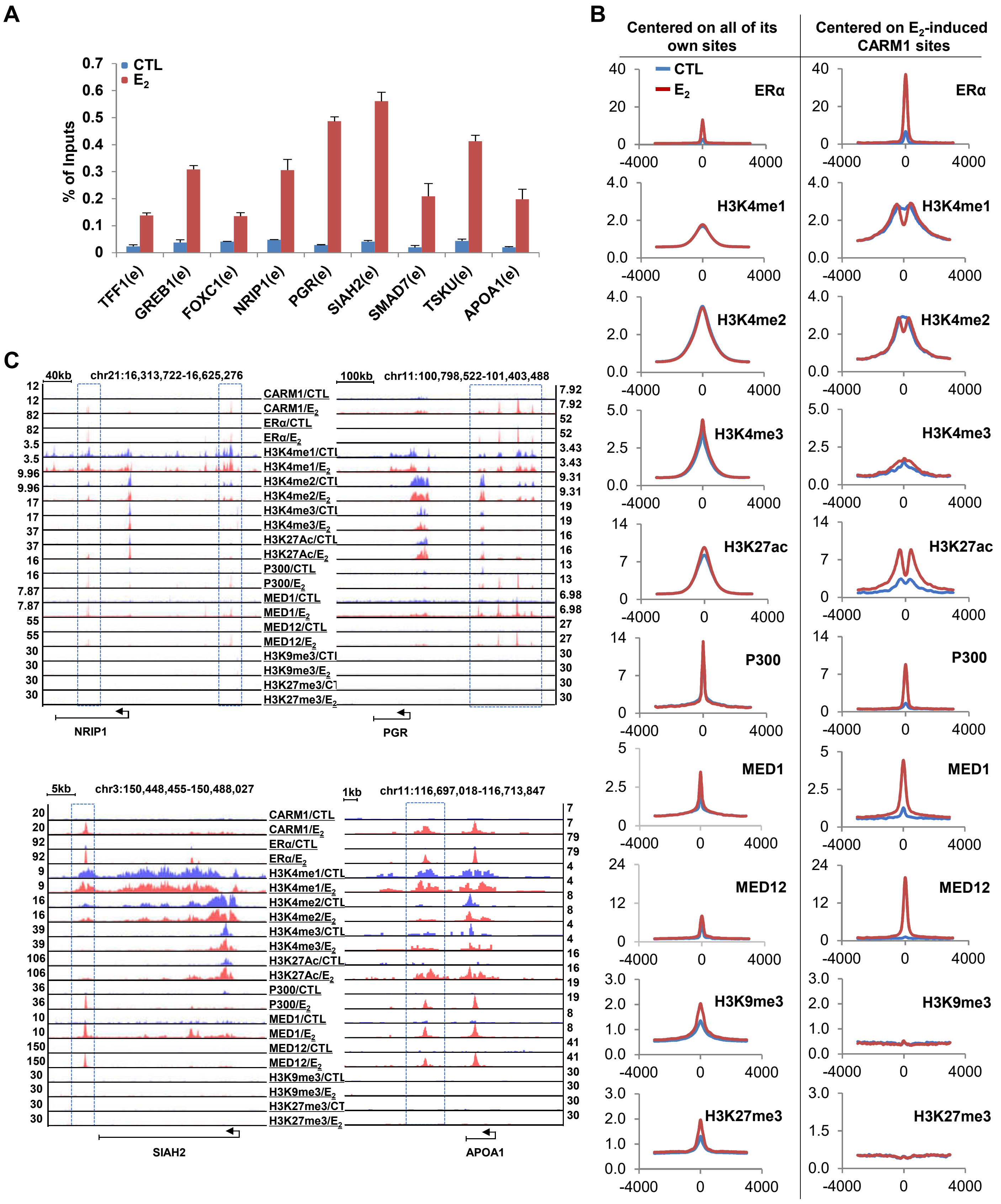
A
% of Inputs
CTL
E2
TFF1(e)
GREB1(e)
FOXC1(e)
NRIP1(e)
PGR(e)
SIAH2(e)
SMAD7(e)
TSKU(e)
APOA1(e)
B
Centered on all of its own sites
Centered on E2-induced CARM1 sites
ERα
H3K4me1
H3K4me2
H3K4me3
H3K27ac
P300
MED1
MED12
H3K9me3
H3K27me3
C
40kb
chr21:16,313,722-16,625,276
100kb
chr11:100,798,522-101,403,488
NRIP1
PGR
5kb
chr3:150,448,455-150,488,027
1kb
chr11:116,697,018-116,713,847
SIAH2
APOA1
CARM1/CTL
CARM1/E2
ERα/CTL
ERα/E2
H3K4me1/CTL
H3K4me1/E2
H3K4me2/CTL
H3K4me2/E2
H3K4me3/CTL
H3K4me3/E2
H3K27Ac/CTL
H3K27Ac/E2
P300/CTL
P300/E2
MED1/CTL
MED1/E2
MED12/CTL
MED12/E2
H3K9me3/CTL
H3K9me3/E2
H3K27me3/CTL
H3K27me3/E2

Figure S2

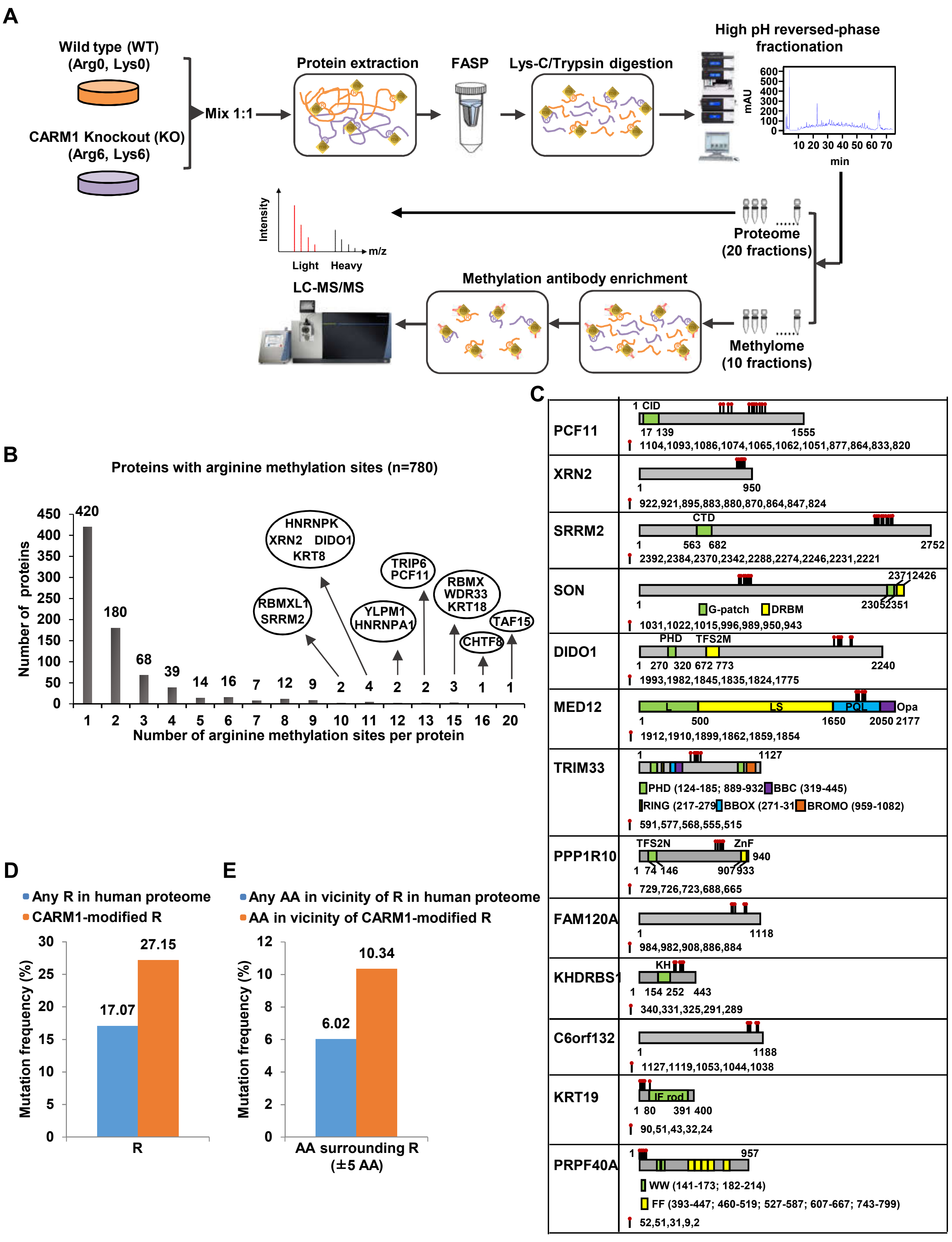
A
Wild type (WT) (Arg0, Lys0)
CARM1 Knockout (KO) (Arg6, Lys6)
Mix 1:1
Protein extraction
FASP
Lys-C/Trypsin digestion
High pH reversed-phase fractionation
mAU
min
Proteome (20 fractions)
Methylome (10 fractions)
Methylation antibody enrichment
Intensity
m/z
Light Heavy
LC-MS/MS
B
Proteins with arginine methylation sites (n=780)
Number of proteins
Number of arginine methylation sites per protein
420 180 68 39 14 16 7 12 9 2 4 2 2 3 1 1
HNRNPK XRN2 DIDO1 KRT8
RBMXL1 SRRM2
YLPM1 HNRNPA1
TRIP6 PCF11
RBMX WDR33 KRT18
CHTF8
TAF15
C
PCF11 1 CID 17 139 1555
1104,1093,1086,1074,1065,1062,1051,877,864,833,820
XRN2 1 950
922,921,895,883,880,870,864,847,824
SRRM2 CTD 1 563 682 2752
2392,2384,2370,2342,2288,2274,2246,2231,2221
SON 1 G-patch DRBM 2371 2426 2305 2351
1031,1022,1015,996,989,950,943
DIDO1 PHD TFS2M 1 270 320 672 773 2240
1993,1982,1845,1835,1824,1775
MED12 L LS PQL Opa 1 500 1650 2050 2177
1912,1910,1899,1862,1859,1854
TRIM33 1 1127
PHD (124-185; 889-932) BBC (319-445)
RING (217-279) BBOX (271-31 BROMO (959-1082)
591,577,568,555,515
PPP1R10 TFS2N ZnF 1 74 146 907 933 940
729,726,723,688,665
FAM120A 1 1118
984,982,908,886,884
KHDRBS1 KH 1 154 252 443
340,331,325,291,289
C6orf132 1 1188
1127,1119,1053,1044,1038
KRT19 IF rod 1 80 391 400
90,51,43,32,24
PRPF40A 1 957
WW (141-173; 182-214)
FF (393-447; 460-519; 527-587; 607-667; 743-799)
52,51,31,9,2
D
Any R in human proteome
CARM1-modified R
Mutation frequency (%)
17.07 27.15
R
E
Any AA in vicinity of R in human proteome
AA in vicinity of CARM1-modified R
Mutation frequency (%)
6.02 10.34
AA surrounding R (±5 AA)

Figure S3

A

MED12 (NP_005111.2) aa1616-2177

LAKKLQKELGERQSDSLEKVRQLLPLPKQTRDVITCEPQGSLIDTKGN
KIAGFDSIFKKEGLQVSTKQKISPWDLFEGLKPSAPLSWGWFGTVRVD
RRVARGEEQQRLLLYHTHLRPRPRAYYLEPLPLPPEDEEPPAPTLLEP
EKKAPEPPKTDKPGAAPPSTEERKKKSTKGKKRSQPATKTEDYGMG
PGRSGPYGVTVPPDLLHHPNPGSITHLNYRQGSIGLYTQNQPLPAGGP
RVDPYRPVRLPMQKLPTRPTYPGVLPTTMTGVMGLEPSSYKTSVYRQ
QQPAVPQGQRLRQQLQQSQGMLGQSSVHQMTPSSSYGLQTSQGYT
PYVSHVGLQQHTGPAGTMVPPSYSSQPYQSTHPSTNPTLVDPTRHLQ
QRPSGYVHQQAPTYGHGLTSTQRFSHQTLQQTPMISTMTPMSAQGVQ
AGVRSTAILPEQQQQQQQQQQQQQQQQQQQQQQQQQYHIRQQQ
QQQILRQQQQQQQQQQQQQQQQQQQQQQQQQQHQQQQQQQAAP
PQPQPQSQPQFQRQGLQQTQQQQQTAALVRQLQQQLSNTQPQPST
NIFGRY

Figure S4

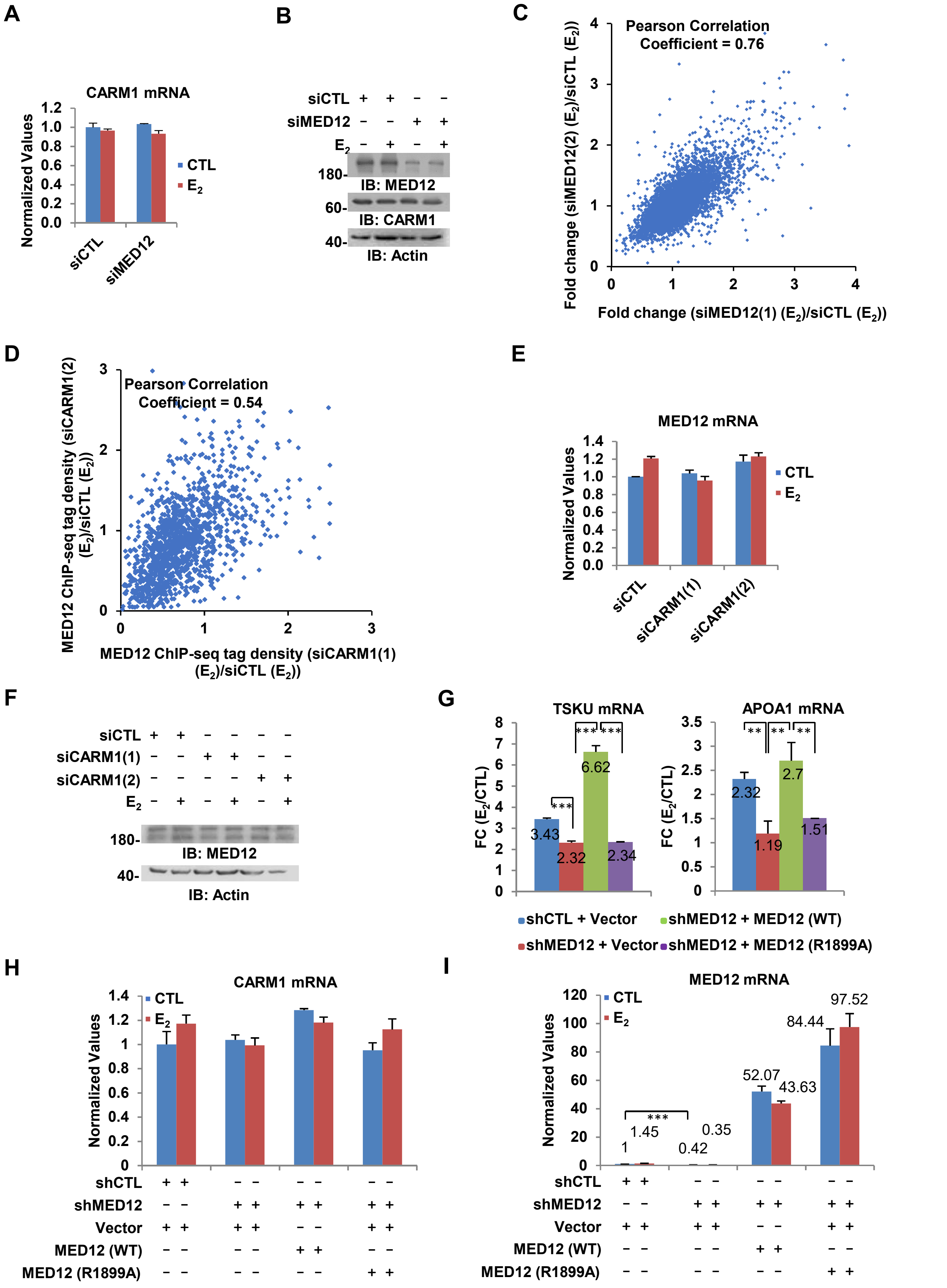
A
CARM1 mRNA
Normalized Values
1.2
1.0
0.8
0.6
0.4
0.2
0.0
CTL
E2
siCTL
siMED12
B
siCTL + + – –
siMED12 – – + +
E2 – + – +
180-
IB: MED12
60-
IB: CARM1
40-
IB: Actin
C
Pearson Correlation
Coefficient = 0.76
Fold change (siMED12(2) (E2)/siCTL (E2))
Fold change (siMED12(1) (E2)/siCTL (E2))
D
Pearson Correlation
Coefficient = 0.54
MED12 ChIP-seq tag density (siCARM1(2) (E2)/siCTL (E2))
MED12 ChIP-seq tag density (siCARM1(1) (E2)/siCTL (E2))
E
MED12 mRNA
Normalized Values
CTL
E2
siCTL
siCARM1(1)
siCARM1(2)
F
siCTL + + – – – –
siCARM1(1) – – + + – –
siCARM1(2) – – – – + +
E2 – + – + – +
180-
IB: MED12
40-
IB: Actin
G
TSKU mRNA
FC (E2/CTL)
3.43
2.32
6.62
2.34
APOA1 mRNA
FC (E2/CTL)
2.32
1.19
2.7
1.51
shCTL + Vector
shMED12 + MED12 (WT)
shMED12 + Vector
shMED12 + MED12 (R1899A)
H
CARM1 mRNA
Normalized Values
CTL
E2
shCTL
shMED12
Vector
MED12 (WT)
MED12 (R1899A)
I
MED12 mRNA
Normalized Values
CTL
E2
1
1.45
0.42
0.35
52.07
43.63
84.44
97.52
shCTL
shMED12
Vector
MED12 (WT)
MED12 (R1899A)

Figure S5

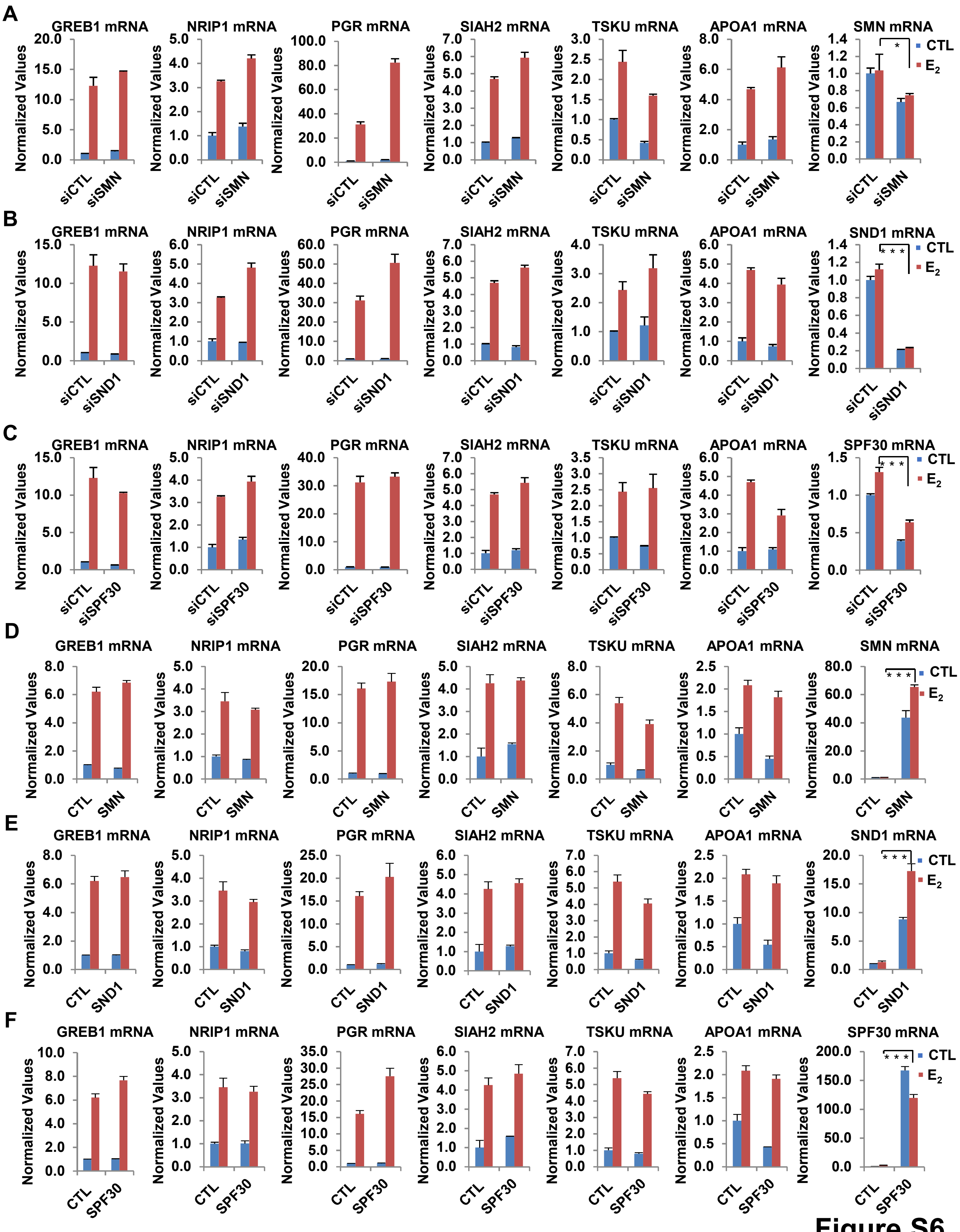
A
B
C
D
E
F
GREB1 mRNA
NRIP1 mRNA
PGR mRNA
SIAH2 mRNA
TSKU mRNA
APOA1 mRNA
SMN mRNA
SND1 mRNA
SPF30 mRNA
Normalized Values
siCTL
siSMN
siSND1
siSPF30
CTL
SMN
SND1
SPF30
CTL
E2

Figure S6

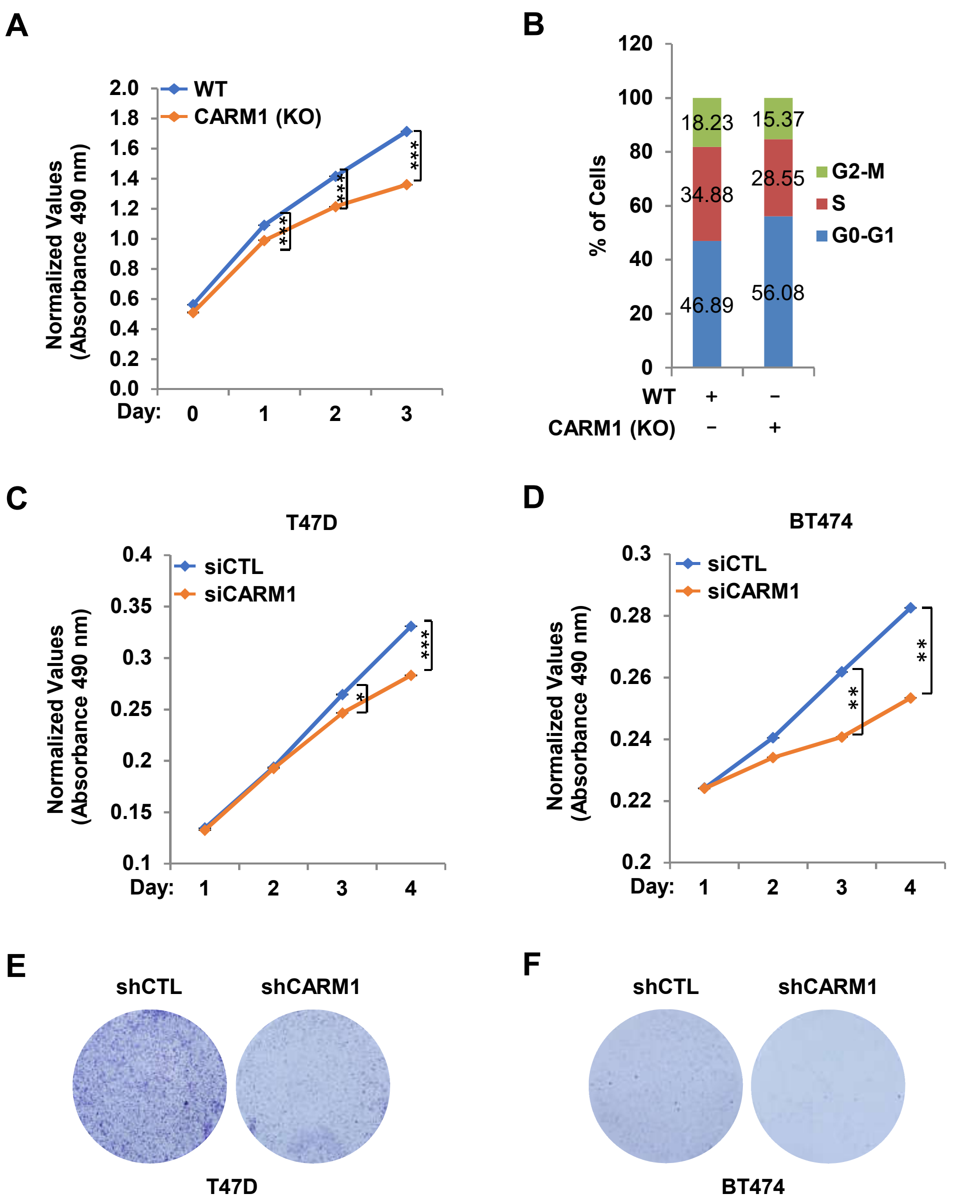
A
WT
CARM1 (KO)
Normalized Values (Absorbance 490 nm)
Day:
***
B
% of Cells
18.23
34.88
46.89
15.37
28.55
56.08
G2-M
S
G0-G1
WT
CARM1 (KO)
C
T47D
siCTL
siCARM1
Normalized Values (Absorbance 490 nm)
Day:
D
BT474
siCTL
siCARM1
Normalized Values (Absorbance 490 nm)
Day:
E
shCTL
shCARM1
T47D
F
shCTL
shCARM1
BT474

Figure S7
